# Amazon Dry Season Will Lengthen Under Future Climate

**DOI:** 10.1111/gcb.71018

**Published:** 2026-07-23

**Authors:** Igor José Malfetoni Ferreira, Nathália S. Carvalho, Lina M. Mercado, Stephen Sitch, Douglas Kelley, Chantelle Burton, Débora Joana Dutra, Scott Barningham, Maria L. F. Barbosa, Julia Mindlin, Celso H. L. Silva‐Junior, Dhruba J. Goswami, Luiz E. O. C. Aragão, Liana O. Anderson

**Affiliations:** ^1^ Remote Sensing Postgraduate Program (PGSER), Coordination for Education, Research and Outreach (COEPE) Brazil's National Institute for Space Research (INPE) São José dos Campos São Paulo Brazil; ^2^ Lancaster Environment Centre Lancaster University Lancaster Lancashire UK; ^3^ Faculty of Environment, Science and Economy University of Exeter Exeter UK; ^4^ Land and Climate System UK Centre for Ecology & Hydrology Wallingford UK; ^5^ Water and Climate System UK Centre for Ecology & Hydrology Wallingford UK; ^6^ Met Office Hadley Centre Exeter UK; ^7^ Leipzig Institute for Meteorology, Leipzig University Leipzig Germany; ^8^ Amazon Environmental Research Institute (IPAM) Brasília Federal District Brazil; ^9^ Federal University of Maranhão (UFMA) São Luís Maranhão Brazil; ^10^ Earth Observation and Geoinformatics Division (DIOTG), Earth Sciences General Coordination (CGCT) Brazil's National Institute for Space Research (INPE) São José dos Campos São Paulo Brazil

**Keywords:** Amazonia, CMIP6, dry season dynamics, earth system models, remote sensing, water deficit, weighted multi‐model ensemble

## Abstract

The Amazon rainforest is a key component of the Earth system, regulating regional climate, sustaining high biodiversity and carbon stocks. However, it is threatened by climate and land‐use changes. In recent decades, the region has experienced intensified droughts and heatwaves, trends expected to worsen under future warming. In this study, we assess how the timing, length, and spatial distribution of the dry season across the Amazon is projected to change this century under different Shared Socioeconomic Pathways. Using a multi‐model ensemble of the Coupled Model Intercomparison Project (CMIP6) weighted according to their RMSE performance, we estimate monthly water balance based on precipitation and evapotranspiration from 2000 to 2100. We calculated dry season onset, end, and length from accumulated water deficits, providing a spatially explicit characterization of seasonal dynamics. We find a significant lengthening of the dry season in 35% of the basin under low‐emission scenarios (SSP1‐2.6) and up to 56% under high‐emission scenarios (SSP5‐8.5), with increases of up to 2 months in the southern and eastern Amazon by 2100. Such changes pose major risks for forest degradation, regional water availability, and climate feedback, potentially reducing the biome's role as a carbon sink. With such large changes even under low emission, our findings demonstrate the urgent need for spatially targeted climate adaptation strategies and mitigation policies that consider future changes in seasonal water availability. While such strategies are essential to enhance societal resilience, safeguarding forest ecosystems and preserving Amazonian hydrological functions ultimately depend on ambitious efforts to reduce greenhouse gas emissions. These actions are essential not only for local and regional sustainability but also for global climate stability.

## Graphical abstract

The Amazon rainforest helps regulate global climate through carbon storage, evapotranspiration, and moisture recycling, yet its future dry season remains uncertain. Using satellite observations and CMIP6 climate model projections, we show that the Amazon dry season is likely to become longer across one‐third of the basin even under low greenhouse gas emissions scenarios (SSP1‐2.6), expanding to more than half of the Amazon under high emissions scenarios (SSP5‐8.5), driven by earlier onset and delayed end of seasonal drought. These changes increase water stress, forest degradation, fire susceptibility, and threaten ecosystem services that affect the livelihoods of millions of people across South America.

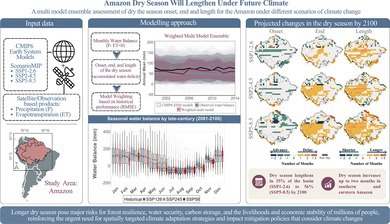

## 1 Introduction

The Amazon rainforest, the largest tropical forest on Earth, harbours unparalleled biodiversity and exerts a profound influence on the Earth's climate system (Fearnside 2008; Malhi et al. 2008; Gatti et al. 2014). It stores an estimated 100–120 Pg of carbon and contributes over 20% of global terrestrial evapotranspiration (Field et al. 1998; Makarieva et al. 2013), functioning as a major carbon sink and a moisture recycling engine for South America (Goetz et al. 2009; Carvalho et al. 2010; Angelini et al. 2013). These processes sustain atmospheric humidity and rainfall across distant regions throughout central and southern Brazil, which depend heavily on Amazon‐derived moisture for agriculture and freshwater security (Staal et al. 2020; Leite‐Filho et al. 2021). However, these ecosystem services are increasingly threatened by anthropogenic climate change, deforestation, and land‐use intensification (Alves et al. 2017; Balch et al. 2022; Reboita and Ambrizzi 2022). Observations and Earth System models consistently show increasing drought and heatwave frequency and intensity across the Amazon (Fu et al. 2013; Marengo et al. 2018; Arias et al. 2023). These climate stressors are expected to worsen under continued global warming, potentially leading to longer and more severe dry seasons (Vogel et al. 2020; IPCC 2021, 2022). Such intensification threatens the region's role as a net carbon sink (Burton et al. 2022, 2024), shifting the Amazon towards a net carbon source by increasing tree mortality and fire risk, and reducing vegetation recovery after following water stress events (Berenguer et al. 2014; Silva Junior et al. 2018; Gatti et al. 2021).

Among the most critical yet understudied outcomes of these pressures are shifting dry season dynamics. Although previous studies have assessed dry season variability, most rely predominantly on precipitation metrics (Duffy et al. 2015; Parsons 2020; Wainwright et al. 2021), overlooking evapotranspiration and the full water balance in modulating seasonal drought stress (Papastefanou et al. 2022). Moreover, dry season length and intensity have typically been calculated assuming a static evapotranspiration threshold of 100 mm/month basin wide, despite recent evidence showing a positive trend in evapotranspiration (Laipelt et al. 2025). This simplification, along with the assumption that the dry period occurs uniformly between August and September, disregards the pronounced spatial and temporal variability of Amazon's water balance (Carvalho et al. 2021). Such omissions limit accurate evaluation of ecological responses, including plant water stress, vegetation productivity, and fire susceptibility (Maeda et al. 2017), thereby undermining national and international climate mitigation and adaptation strategies, including Brazil's commitments under the Nationally Determined Contributions (NDCs). Addressing this gap is essential for improving projections of future Amazon droughts. Considerable uncertainty remains regarding future spatial and temporal dry season patterns, particularly given the basin's ecological and climatic heterogeneity. These uncertainties include potential nonlinear ecological responses to prolonged hydrological stress, with some earth system models indicating increasing risk of drought‐induced tree mortality and large‐scale ecosystem degradation (Nobre et al. 2016; Yao et al. 2024).

Addressing these multifaceted challenges requires robust, long‐term monitoring. In this regard, remote sensing has become essential for tracking key climate variables such as evapotranspiration, precipitation, and vegetation status across large areas. However, individual satellite products differ in temporal resolution, sensor characteristics, and retrieval algorithms, constraining the accuracy of water balance estimates (Miralles et al. 2016; Martens et al. 2017). Therefore, integrating multiple observational datasets helps to reduce uncertainties and improve the detection of climate‐driven ecosystem changes (Mu et al. 2011a; Martens et al. 2017; Pan et al. 2012). These observational datasets are also fundamental for evaluating and constraining Earth System Models (ESMs), providing a critical link between observational and model‐based projections.

In the Amazon context, climate change scenarios provide a useful framework for evaluating how warming‐driven shifts in seasonal hydrology, such as changes in dry season onset, end, and length, may unfold in the coming decades to support regional adaptation and mitigation planning (Riahi et al. 2017). However, ESMs vary in how they simulate key processes, including physical parameterisations, spatial resolution, and climate sensitivity, often generating divergent outcomes. To address these structural uncertainties, multi‐model ensembles has become a widely adopted strategy to enhance the robustness of future climate projections, particularly in regions with complex ecological and climatic feedbacks like the Amazon (Tebaldi and Knutti 2007; Gulizia and Camilloni 2015; Knutti et al. 2017; Tebaldi et al. 2021). These complexities make the system difficult to model accurately. Weighting methodologies have therefore been proposed to aggregate ensemble projections by assigning greater influence to models that better reproduce observed climate conditions, under the assumption that historical performance reflects future predictive skill. Although widely accepted, this assumption remains a limitation, particularly if future climate responses diverge from historical patterns. The latest generation of ESMs from the Coupled Model Intercomparison Project Phase 6 (CMIP6) simulate atmospheric, oceanic, terrestrial, and biogeochemical processes, including land use and vegetation changes (Boysen et al. 2020; Jones 2020; IPCC 2022). Within CMIP6, the Scenario Model Intercomparison Project (ScenarioMIP) provides coordinated experiments integrating Shared Socioeconomic Pathways (SSPs) with Representative Concentration Pathways (RCPs), enabling exploration of plausible range of future trajectories shaped by socioeconomic development and emissions trends (O'Neill et al. 2016; Riahi et al. 2017; IPCC 2022). While CMIP6 defines the modeling framework, ScenarioMIP outlines the range of socioeconomic and policy pathways through which climate change may unfold. These scenarios span from the sustainable, high mitigation, low‐emissions SSP1‐2.6 (limiting warming to approximately 1.8°C by 2100), through the intermediate‐mitigation efforts SSP2‐4.5 (warming of around 2.7°C), representing moderate emission and consistent with current policies and technological trends, to no mitigation, high‐emissions, business as usual SSP5‐8.5, which projects warming above 4°C by the end of the century (Fricko et al. 2017; Kriegler et al. 2017; Riahi et al. 2017; van Vuuren et al. 2017).

Although some studies have provided spatially explicit projections of dry season change (Parsons 2020; Ukkola et al. 2020; Yao et al. 2024), an integrated approach that combines precipitation with spatially and temporally variable evapotranspiration, using a weighted multi‐model ensemble to reduce uncertainty, is still lacking for the entire Amazon. Therefore, this study aims to project changes in timing, length, and spatial distribution of the Amazon dry season throughout the 21st century under three SSP scenarios (SSP1‐2.6, SSP2‐4.5, SSP5‐8.5). To support this analysis, we first evaluate the ESM performance in simulating historical water balance dynamics by comparing their outputs against satellite‐based observational datasets. Based on this evaluation, a performance‐based weighting scheme is applied to a set of 10 CMIP6 models to enhance robustness of future projections, acknowledging that this approach assumes continuity between historical model performance and future skill. We then analyze changes for mid‐century (2041–2060) and late‐century (2081–2100). Finally, we discuss our results through the lens of how dry season changes may affect broader ecological, hydrological, and social systems, and explore implications for national policies, providing critical insights for adaptation strategies and long‐term ecosystem sustainability in a changing climate.

## 2 Material and Methods

We first use observational precipitation and evapotranspiration products (Section 2.2.1) to evaluate the spatial water balance, providing a benchmark to quantify biases and uncertainties in the simulated water balance. ESM outputs for historical and future simulations are then described (Section 2.2.2), followed by the description of the approach for identifying dry season onset, end, and length (Section 2.3). Model performance and related uncertainties are assessed through Root Mean Square Error and absolute mean bias metrics (Section 2.4), and a performance‐based weighting scheme is applied to enhance ensemble reliability (Section 2.5). Results are organized into three parts: ESM performance in representing annual mean water balance relative to observations (Section 3.1), spatial and temporal patterns of the observed and simulated historical dry season (Section 3.2), and projected future changes under different emission scenarios (Section 3.3). The discussion focuses on model performance (Section 4.1), potential impacts of future dry season changes (Section 4.2), and the main limitations and uncertainties of the models used (Section 4.3).

### 2.1 Study Area

**FIGURE 1:**
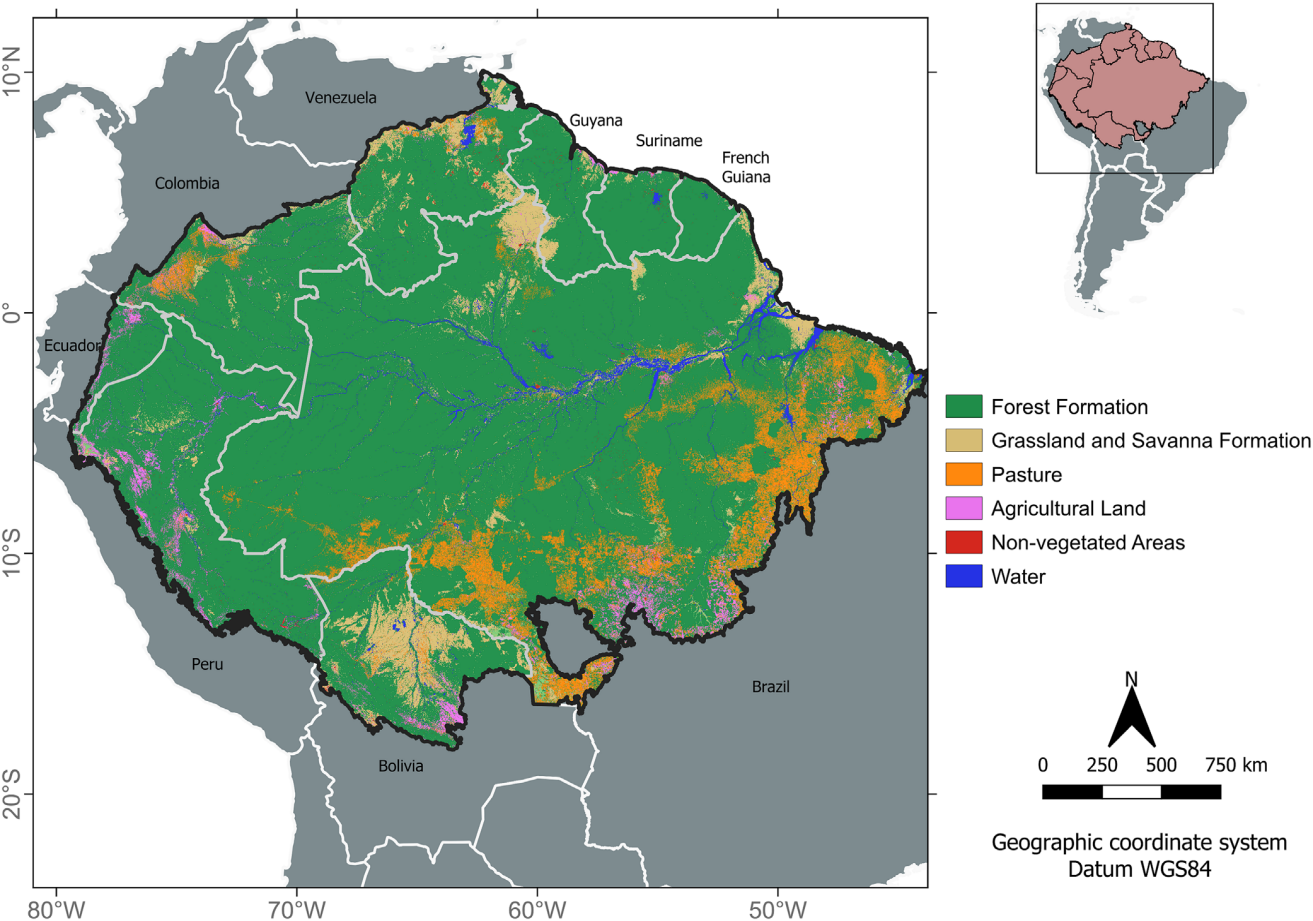
Location of Amazonian Biome in South America. Map lines delineate study areas and do not necessarily depict accepted national boundaries.

The study area encompasses the Amazon Basin, covering ~6.7 million km^2^ across nine South American countries (Figure 1). Brazil contains 62% of this area (MapBiomas Amazônia 2022), comprising most edaphic and climatic conditions. The remainder spans Peru (11%), Bolivia (8%), Colombia (6%), Venezuela (5%), Guyana (3%), Suriname (2%), Ecuador (2%), and French Guiana (1%). The landscape includes dense and open tropical forests (~30% of the world's total), while wetlands, grasslands and savannas together cover the remaining approximately 21% of the basin total area (Food and Agriculture Organization of the United Nations 2005; MapBiomas Amazônia 2022). Precipitation is spatially and seasonally variable, averaging 2200 mm annually and exceeding 5,000 mm in some regions. Recurrent extreme droughts linked to positive anomalies in tropical sea surface temperatures such as El Niño also affect the region (Espinoza Villar et al. 2009).

### 2.2 Datasets

#### 2.2.1 Observational Data

In this study, remote sensing products serve as independent observational datasets for characterizing historical conditions and as benchmarks for evaluating Earth System Model simulations, thereby enhancing the robustness of model‐based projections. To represent observed conditions (ground‐truth), we employed monthly precipitation data from Climate Hazards Group InfraRed Precipitation with Station‐CHIRPS (Funk et al. 2015) and five evapotranspiration (ET) datasets across the Amazon from 2000 to 2014 (Appendix S1 and Table S1). CHIRPS integrates satellite and ground station data into a consistent grid, suitable for trend analysis (Funk et al. 2015), and it explains up to 73% of the variance in rain gauge data in the Amazon (Anderson et al. 2018). The evapotranspiration datasets include MOD16A2GF, which utilises MODIS data and the Penman–Monteith equation (Mu et al. 2011b); GLDAS and FLDAS, combining satellite and ground observations (Rodell et al. 2004; McNally et al. 2017); GLEAM, estimating land evaporation and soil moisture based on remote sensing inputs (Miralles et al. 2011); and TerraClimate, offering monthly water balance variables using the Penman–Monteith approach (Abatzoglou et al. 2018). These observational datasets were used as reference to evaluate the ESM performance in simulating water balance across the region (see Section 2.4).

#### 2.2.2 Earth System Model Output

We utilized precipitation and evapotranspiration output from 10 CMIP6 ESMs (Tables S2 and S3) for the historical baseline (2000–2014) and future projections for mid‐century (2041–2060) and late‐century (2081–2100) under SSP1‐2.6, SSP2‐4.5, and SSP5‐8.5 scenarios, encompassing a broad range of socio‐economic and emissions pathways (Appendix S2). Model selection prioritized the availability of consistent ensemble member variants across variables and scenarios, as well as a native horizontal resolution near 1° (approximately 111 km). This procedure minimizes the discrepancies from ensemble‐specific variability and adequately represents mesoscale processes, such as convective systems, river breezes, and subtle topographic variations, that are critical for simulating the spatial and temporal variability of precipitation and evapotranspiration in the Amazon (Silva et al. 2023; Tai et al. 2024). To ensure comparability with satellite‐derived observations and the onset of ScenarioMIP projections, we defined the baseline as the 2000–2014 period.

### 2.3 Identifying the Onset, End, and Length of Dry Season

We resampled all datasets to a common spatial resolution of 1° to ensure consistency in spatial analysis before applying the bilinear interpolation method, which estimates values as a weighted average of the four nearest neighboring grid cells. This produces a smooth transition, preserving spatial gradients and is preferred for precipitation and evapotranspiration because it minimizes artificial discontinuities when using continuous variables based on mean values resampling methods, and balances computational efficiency and spatial accuracy (Burrough and McDonnell 1998; Hijmans et al. 2005; Li and Heap 2014).

We evaluated dry season variation by estimating the water balance for each grid cell as the difference between precipitation and evapotranspiration (Figure S1). At each grid cell, the dry season was identified as one or more consecutive months when evapotranspiration exceeds precipitation, resulting in a water deficit, following Carvalho et al. (2021). The length, onset, and end of the dry season were mapped to analyze spatiotemporal changes between baseline and future scenarios.

For the observed baseline, we used CHIRPS precipitation and the multi‐product evapotranspiration mean to calculate the water balance. To account for regional hydrological variability and limited reference data (Aragão et al. 2008; Maeda et al. 2017), we computed a multi‐ensemble mean of five evapotranspiration satellite‐based products (Table S1). This approach reduces the influence of individual product‐specific biases and partially constrains uncertainty arising from differences in evapotranspiration parameterizations (Maeda et al. 2017; Derardja et al. 2024). However, satellite‐based evapotranspiration products remain model‐derived estimates, and their spread largely reflects differences in parameterization rather than independent observational uncertainty (Miralles et al. 2016; Martens et al. 2017).

For the historical and future simulations, we applied this method to each ESM's ensemble member. Additionally, we calculated changes in the distribution and length of the dry season as the differences between the historical simulated period (2000–2014) and the projected dry season under each scenario (SSP1‐2.6, SSP2‐4.5, and SSP5‐8.5) for mid‐ and late‐century.

### 2.4 Earth System Model Evaluation and Uncertainty

We assess the performance of historical ESM simulations in reproducing observed water balance dynamics across the Amazon using two quantitative metrics: the Root Mean Square Error (RMSE) and the absolute mean bias (AMB), and one categorical comparison between observed and simulated dry conditions. The RMSE was calculated monthly by comparing the observed water balance with the simulated historical water balance from each ESM. We computed RMSE for each ensemble member of every model, considering the mean over the time period from 2000 to 2014 and a spatially averaged RMSE value for the entire Amazon (Equation 1).

(1) $$\text{RMSE}=\sqrt{\frac{1}{n}\sum_{i=1}^{n}{\left({\mathrm{WB}}_{\mathrm{sim}}\left(n\right)-{\mathrm{WB}}_{\mathrm{obs}}\left(n\right)\right)}^{2}}$$

where 𝑛 is the total number of months, ${\mathrm{WB}}_{\mathrm{sim}}$ and ${\mathrm{WB}}_{\mathrm{obs}}$ represent the simulated and observed mean water balance value over the time period, respectively, for each grid cell.

The AMB was computed for each model and ensemble member to evaluate the spatial performance of simulated water balance against observed values. This metric highlights whether models systematically overestimate or underestimate the water deficit across the region and provides a direct measure of the spatial accuracy of each simulation. The mean bias was calculated as the absolute difference between simulated and observed values (Equation 2). By incorporating RMSE and mean bias metrics, our analysis evaluates the magnitude of errors (via RMSE) and the directional accuracy (via AMB) in model simulations.

(2) $$\mathrm{AMB}=\frac{1}{n}\sum_{i=1}^{n}\left|{\mathrm{WD}}_{\mathrm{sim}}\left(n\right)-{\mathrm{WD}}_{\mathrm{obs}}\left(n\right)\right|$$

Additionally, we evaluated the spatial accuracy of dry season classification using a categorical comparison between observed and simulated dry conditions for each month. In this analysis, false positives refer to grid cells where the model incorrectly classified a wet region as dry, while false negatives indicate cells where the model failed to detect a dry region that was observed. These classification errors are useful for identifying spatial mismatches in the simulation of seasonal drought patterns.

To assess statistical differences in the distribution of annual mean water balance across CMIP6 models, we performed the Kruskal‐Wallis test, a non‐parametric method suitable for comparing more than two groups. This analysis allows us to identify whether models produce statistically distinguishable climatologies, thereby providing insight into inter‐model variability and potential structural biases. Additionally, we applied Dunn's post hoc test with Bonferroni correction for pairwise comparisons when significant differences (*p* ≤ 0.05) were found.

The proposed combination of assessment metrics provides a foundation for interpreting the reliability of future projections and their implications for understanding changes in the dry season distribution and intensity across the Amazon.

### 2.5 Multi‐Model Weighting

Individual ESMs vary significantly in their ability to replicate observed mean climate variables and respective trends, often due to structural limitations and inherent biases within the models (Gleckler et al. 2008; Knutti and Sedláček 2013). To address this issue, we applied a tailored weighting scheme to account for individual model performance, ensuring that better‐performing models contribute more to the final ensemble. The weighting methodology followed a similar approach outlined by Burton et al. (2024) and Knutti et al. (2017). We assigned weights to individual models based on their deviation from the observed water balance according to Equation (3).

(3) $$w_{i}=\frac{e^{-D_{\frac{i}{\sigma_{D}}}}}{E_{i}}$$

where $w_{i}$ represents the weight assigned to model i, $D_{i}$ is the observational distance calculated as the monthly RMSE of each model. $E_{i}$ denotes the number of ensemble members for that model, and $\sigma_{D}$ is the shape parameter that controls the sensitivity of the weighting to model performance. A large constant $\sigma_{D}$ results in nearly equal weights across models (“democratic weighting”), whereas smaller values concentrate the weights on better‐performing models. We adopted $\sigma_{D}$ equal to 0.5, which offers a balanced compromise between democratic weighting and performance‐driven weighting (Burton et al. 2024).

This methodology is particularly advantageous as it accommodates varying numbers of ensemble members across models. It effectively mitigates the overrepresentation of models with more ensemble members by treating these as duplicate contributions and applying appropriate down‐weighting (Knutti et al. 2017). Additionally, the calculated weights were normalized and computed separately for each month to reflect temporal variability.

The final weighted ensemble water balance was calculated by applying the weights to the monthly simulated values from each model, as defined in Equation (4).

(4) $${\mathrm{WB}}_{\text{weighted}}=\sum_{i=1}^{N}w_{i}x\;{\mathrm{WB}}_{\mathrm{sim},i}$$

where ${\mathrm{WB}}_{\text{weighted}}$ is the weighted ensemble water balance, ${\mathrm{WB}}_{\mathrm{sim},i}$ represents the simulated water balance for model i.

The same methodology was consistently applied to calculate the weighted projections of water balance for each scenario and time period. This approach ensures that model performance and ensemble variability are robustly accounted for in both historical and future simulations.

To assess whether median differences in water balance between the historical and future projections were statistically significant, we applied the paired Wilcoxon signed‐rank test (W statistic), as our data is non‐normally distributed and spatially paired. In addition, differences among future estimates across emission scenarios (SSP1‐2.6, SSP2‐4.5, and SSP5‐8.5) were tested using the Kruskal‐Wallis non‐parametric test. Statistical significance for both tests was assessed at the 95% confidence level (*p*‐value ≤ 0.05).

## 3 Results

### 3.1 Earth System Model Performance: Mean Annual Water Balance

**FIGURE 2:**
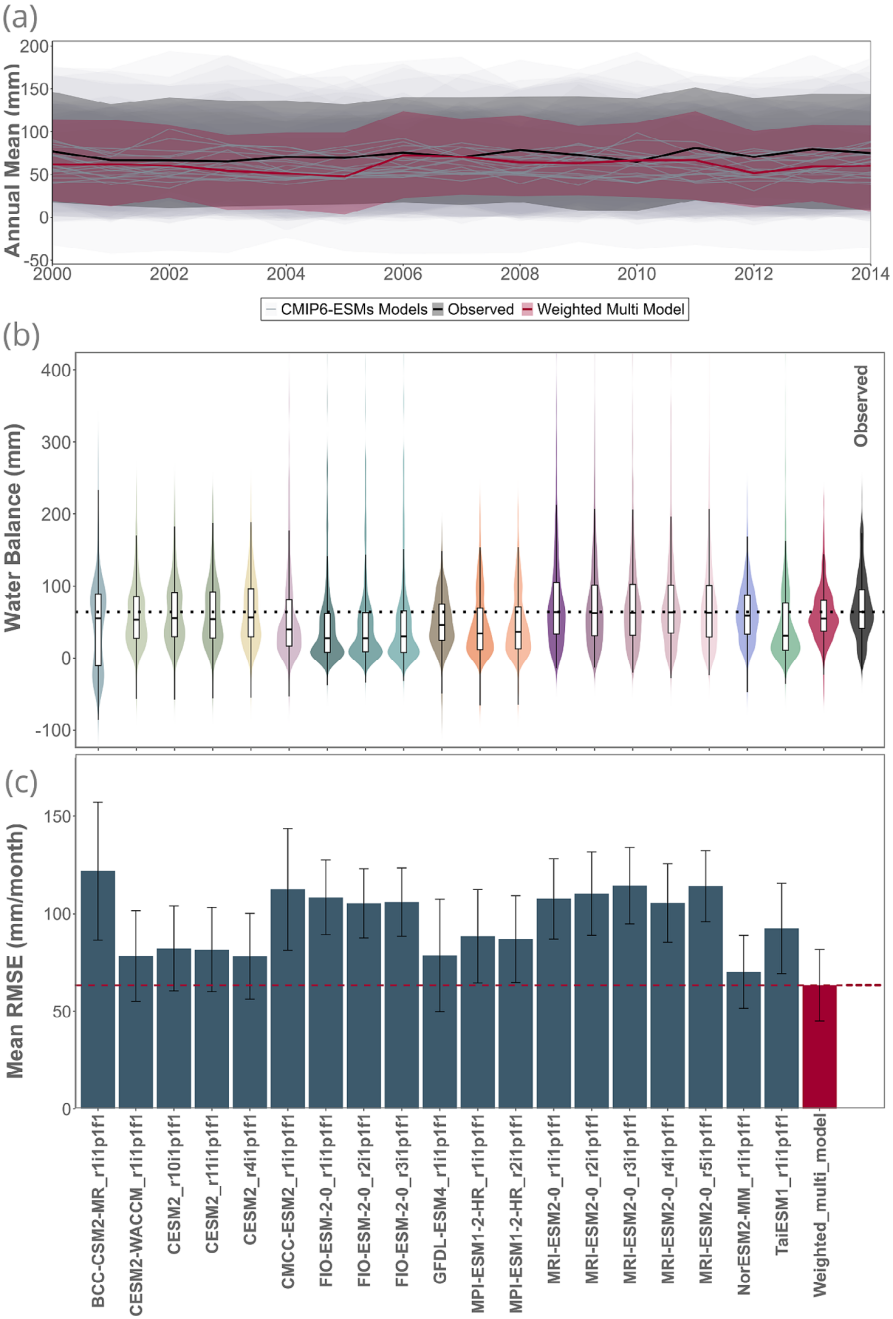
Observed and simulated water balance over the Amazon Basin during the historical period (2000–2014) using CMIP6 models. (a) Annual mean time series: black line represents the observations, red line the weighted multi‐model mean, gray and red shaded areas indicate the 10th–90th percentile range of observed and weighted multi‐model variability, respectively. Gray lines show the individual CMIP6‐ESM simulations. (b) Distribution of mean annual water balance for each individual CMIP6‐ESM simulation, the weighted multi‐model ensemble, and observations. Boxes represent the interquartile range (25th–75th percentile) of the annual mean, whiskers extend to 1.5 times the interquartile range, and the black dotted line marks the observed median. (c) Mean RMSE of each CMIP6 model compared to observations for historical period. Bars represent the mean RMSE (mm/month) for each model variant, with black vertical lines indicating the standard deviation.

Observed and simulated water balance estimates differ in magnitude and interannual variability during 2000–2014 (Figure 2). The observed annual mean deficit was approximately 72 ± 8 mm(Figure 2a), whereas the weighted multi‐model mean was approximately 60 ± 13 mm. Most CMIP6‐ESM simulations fall within the observed interquartile range, which also presents large variability and highlights the high spatial heterogeneity of observed water balance across the basin. Additionally, individual model outputs vary widely and show strong skewness. We reported median deficits ranging from 20 to 80 mm and some simulations exceeding 200 mm (Figure 2b). Conversely, the ensemble median (~55 mm) is closer to observations (64 mm), indicating improved consistency with observations.

Statistical analysis using the Kruskal‐Wallis and Dunn's post hoc test has confirmed significant differences among several models (Figure S2). Model performance, as measured by RMSE, also varied considerably (Figure 2c), indicating differences in their ability to reproduce observed hydrological conditions. BCC‐CSM2‐MR showed the highest RMSE, exceeding 121 ± 35 mm/month, which reflects substantial deviations from observed water balance patterns. In contrast, models such as CESM2 and Nor‐ESM2‐MM show lower RMSE values, around 80 ± 22 mm/month, suggesting relatively better performance. The weighted multimodel ensemble (red bar) consistently outperforms individual models, achieving the lowest RMSE (~63 ± 18 mm/month). Moreover, its narrower error bars highlight reduced uncertainty compared to the wide inter‐model spread observed in individual simulations, underscoring the robustness of the ensemble approach (Figure 2b).

### 3.2 Spatial and Temporal Distribution of Amazon Dry Season

**FIGURE 3:**
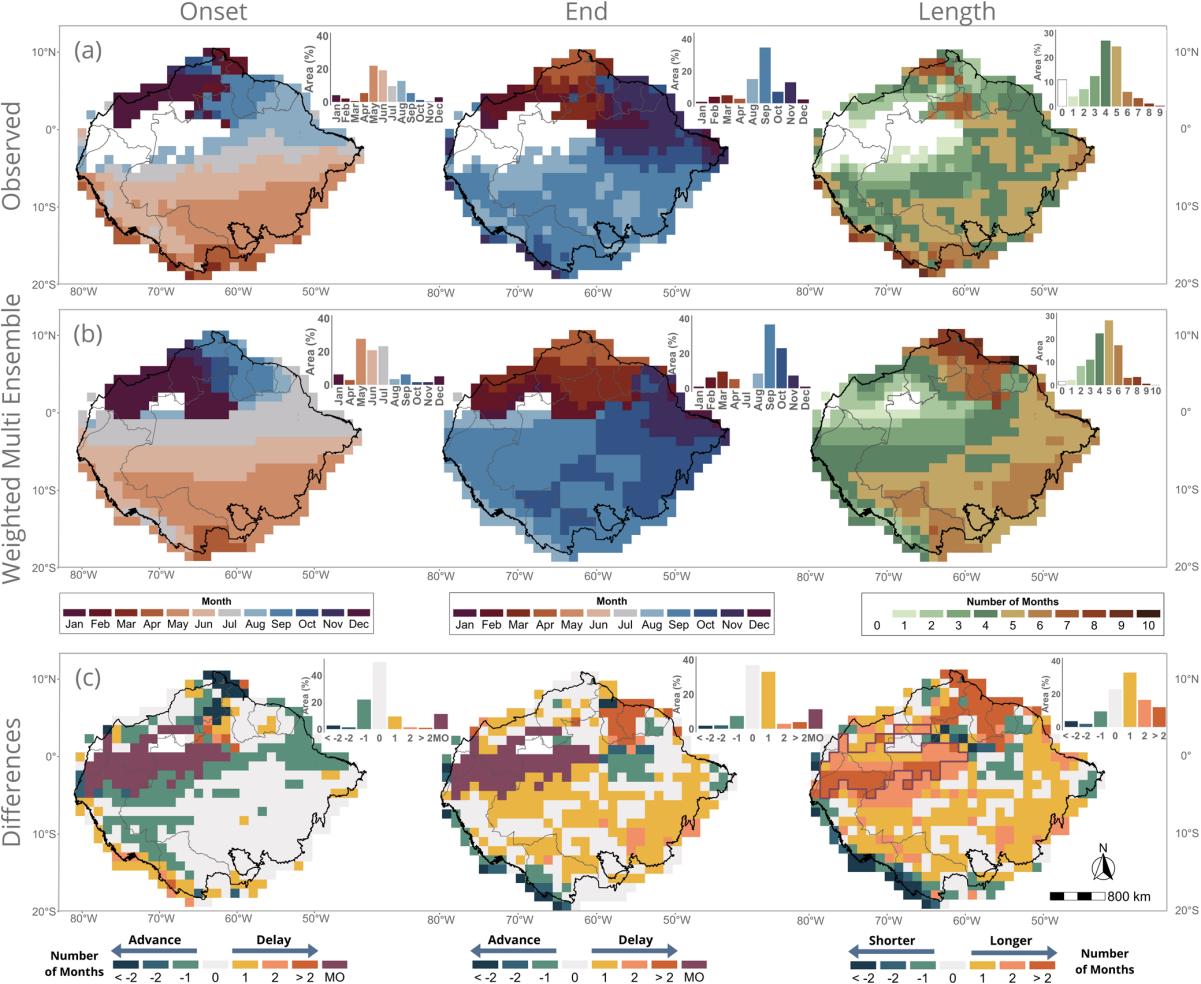
Spatial and temporal distribution of the dry season onset, end, and length across the Amazon during the historical period (2000–2014). (a) Spatial and temporal distribution of the dry season patterns from satellite observations. (b) Simulated dry season patterns from the weighted ESM ensemble. (c) Differences between the simulated and observed dry season patterns, where positive values indicate a delayed onset or end, and a longer dry season length, whereas negative values represent an earlier onset or end, and a shorter dry season length; MO denotes model‐only occurrence of dry season. Map lines delineate study areas and do not necessarily depict accepted national boundaries.

The weighted ensemble captures the overall spatial dynamics of observed water balance across the Amazon although misclassification tends to occur in transitional forest zones and during seasonal shifts (Figure 3). These discrepancies are mainly associated with either delays or early predictions in the timing and spatial extent of drought (see Figure S3 for detailed evaluation of monthly errors and spatial bias patterns).

The dry season variability estimated from satellite observations follows a clear latitudinal gradient, reflecting opposing seasonal patterns between Northern and Southern Hemispheres (Figure 3). Dry season onset varies from January to July, with most of the Amazon beginning in May (22%) and June (19%), especially in central and southern areas (5° S–20° S, Figure 3a and Table S4). The dry season ends predominantly between August and November, with 34.9% of the region ending in September, followed by August (15%) and November (13%). In the Northern Amazon (5° N–5° S), patterns diverge as the west (16% of the region) experiences drought from December to April, while in the east (23% of the region) from August to December. Dry season length varies between three and 7 months across most of the biome, with longer dry periods concentrated along the southern boundary and parts of the northern Hemisphere (Figure 3a and Table S4). On average, a 4 to 5‐month dry season is observed in 27% and 25% of the area, respectively, while regions where the dry season lasts more than 7 months are less common, covering less than 5% of the total basin (Table S4). In the northwestern Amazon, near the equator, 12% of the basin experiences a short dry season lasting only one or 2 months, while 13% of the biome does not exhibit a well‐defined dry season (Figure 3a). These areas are spatially close but distinct, with the short‐season zones typically surrounding the regions with no defined seasonality.

The ESM ensemble captures the spatial and temporal patterns of the dry season, closely resembling observations but showing regional differences (Figure 3b). Overall, the simulated onset aligns with observations in 50% of the biome, but it simulates an earlier onset by 1 month across central‐northern and parts of the southern Amazon, and a delayed onset in the northwest and southwest (Figure 3c). The ESM ensemble also suggests that the dry season predominantly begins in May and June across the Amazon, as suggested by the satellite observations. However, it overestimates the extent of areas where the dry season starts in July, simulating 23% compared to the 10% observed (Table S4).

The ESM ensemble simulates a one‐month delay in the end of dry season across central‐southern areas, and one‐month advance along the northeastern and southwestern boundaries. The simulated end aligns with observations in 37% of the biome, while most of the biome (53%) is simulated with a delayed end of at least 1 month. In both the observations (35%) and the ESM ensemble (36%), September marks the end of the dry season for most of the southern Amazon. However, the ESM ensemble also underrepresents areas with no dry season, simulating only 2% of the Amazon versus 13% observed. These biases lead to an overestimation of dry season length in 62% of the biome, mostly by 1 month (33%). Some regions are simulated with up to 10 months of drought, exceeding the nine‐month maximum observed. Nonetheless, the ESM ensemble accurately simulates the dry season length in 23% of Amazon and similarly captures 4‐ to 5‐month dry seasons in 23% and 29% of the region, respectively.

### 3.3 Projecting Dry Season Dynamics

**FIGURE 4:**
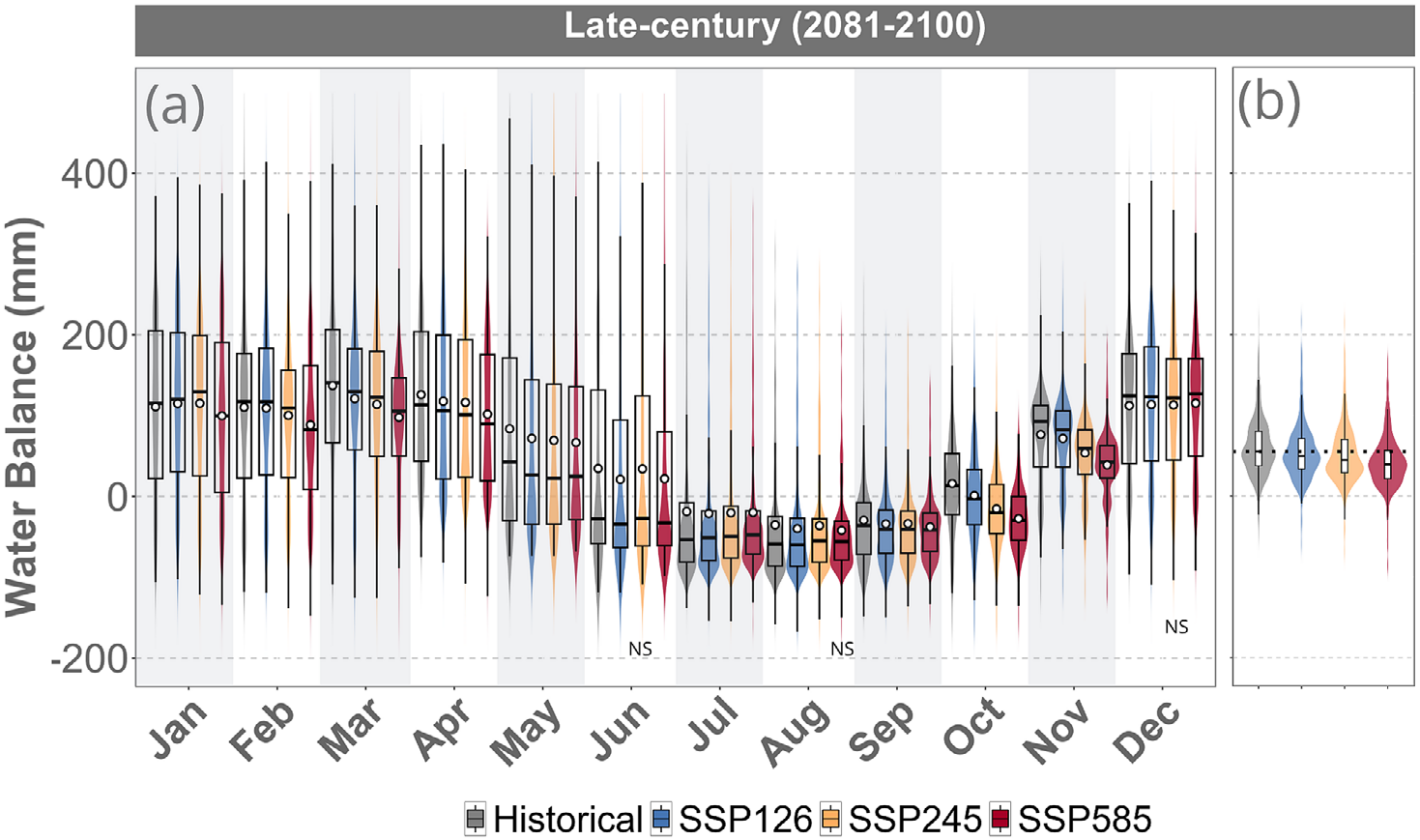
(a) Monthly water balance distribution across the Amazon for the historical period (2000–2014) and future projections under SSP1‐2.6, SSP2‐4.5, and SSP5‐8.5 for late‐century (2081–2100). (b) Annual mean water balance distribution. Each box represents the interquartile range (25th–75th percentile) of the monthly water balance, with whiskers extending to 1.5 times the interquartile range. White points indicate mean value. All scenarios differ significantly (*p*‐value < 0.05), except for the months marked with NS (Non‐Significant).

The projected monthly water balance distribution across the Amazon shows an average seasonal pattern, with the driest months occurring between June and September across all scenarios (Figure 4a). Future projections indicate progressively more negative water balance conditions relative to the historical period (2000–2014). Changes are already detectable by mid‐century (2041–2060), indicating an early tendency toward drier conditions across parts of the basin. However, these shifts are generally smaller in magnitude and less spatially consistent across scenarios and models than those projected for late‐century (2081–2100). By late‐century, the drying signal becomes substantially stronger and more spatially coherent, suggesting a progressive increase in seasonal drought conditions under continued warming.

Among climate and socioeconomical pathways, SSP1‐2.6 presents the smallest deviation from historical conditions, with median values 10% lower than in the historical (Figure 4b). SSP2‐4.5 indicates a moderate reduction of about 19%, reflecting more negative water balance conditions, while SSP5‐8.5 projects the strongest intensification, with median values 34% lower (i.e., higher deficit). By late‐century, the Amazon's water balance under SSP5‐8.5 is projected to deviate by at least 22% from that under SSP1‐2.6 and SSP2‐4.5, indicating a greater likelihood of drier conditions. Although the seasonal timing remains largely unchanged, the magnitude of negative water balance increases with emission levels, indicating more extreme conditions under higher‐emission pathways.

These changes in both onset and end of the dry season lead to a lengthened dry season, mostly by 1 month, across southern and eastern Amazon. Under SSP1‐2.6, 35% of the area experiences a prolonged dry season, while under SSP2‐4.5 it extends to 40%. The most severe changes occur under SSP5‐8.5, with 56% of the region experiencing a prolonged dry season of at least 1 month. Additionally, approximately 50,000 km^2^ presently simulated without dry season will be exposed to at least 1 month of dryness under SSP5‐8.5, a reduction of 36% (Figures S4 and S5).

**FIGURE 5:**
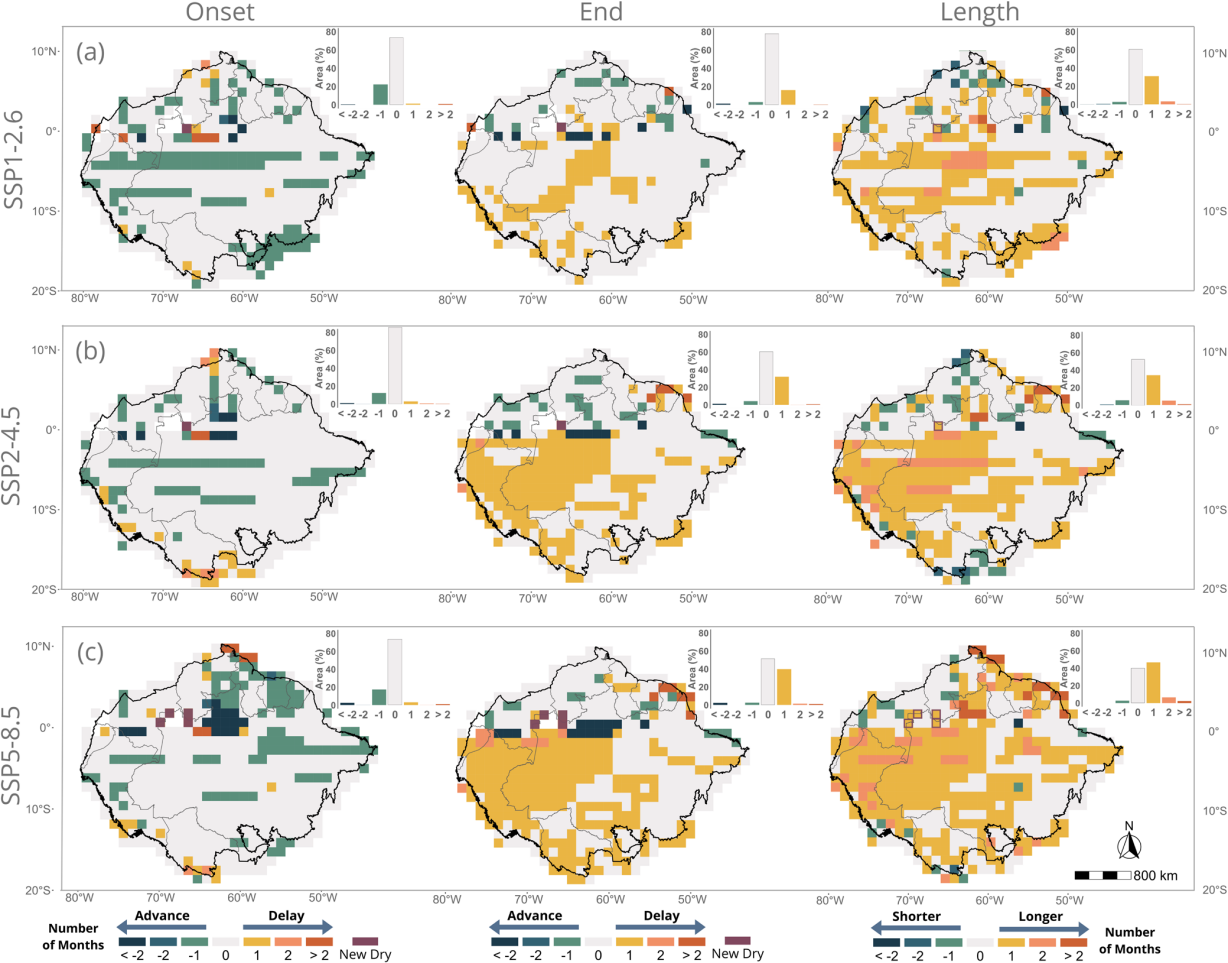
Spatial changes in the onset, end, and length of the dry season in the Amazon simulated by the weighted multi‐model ensemble mean for the late‐century (2081–2100) under the (a) SSP1‐2.6, (b) SSP2‐4.5, and (c) SSP5‐8.5 climate scenarios, across compared to the historical period simulation from 2000 to 2014. Positive values indicate a delayed onset or end, and a longer dry season length, while negative values represent an earlier onset or end, and a shorter dry season length. All scenarios were significantly different (*p*‐value < 0.05). Map lines delineate study areas and do not necessarily depict accepted national boundaries.

By late‐century, changes in the onset, end, and length of the dry season in the Amazon are simulated under the SSP1‐2.6, SSP2‐4.5, and SSP5‐8.5 scenarios (Figure 5). Moderate shifts emerge by mid‐century (Figure S6 and Table S5), but the strongest changes are expected to occur by late‐century. All three scenarios indicate an earlier onset, especially under SSP1‐2.6 affecting 24% of the region (23% experiencing up to 1 month earlier). In this scenario, the longer dry season mainly results from advances in the dry season onset. Under SSP5‐8.5, more extreme advances (over 3 months) affect 3% of the region, mostly in the northern Amazon (Figure 5c). Conversely, all scenarios project a progressive delayed end, with affected areas increasing from 17% under SSP1‐2.6 to 33% in SSP2‐4.5 and 43% in SSP5‐8.5. In SSP2‐4.5 and SSP5‐8.5, the longer dry season is primarily driven by this delayed end. The most impacted area (40% under SSP5‐8.5) experiences up to 1 month of delay, mainly across the southwestern Amazon, while areas with earlier endings remain concentrated to the north, covering less than 6% of the total region.

All pairwise comparisons between the historical baseline and future projections showed statistically significant median shifts in water balance (paired Wilcoxon signed‐rank test, *p*‐value < 0.05) (Tables S6 and S7). Likewise, differences among future projections across emission scenarios were significant for all cases evaluated (Kruskal–Wallis test, *p*‐value < 0.05).

## 4 Discussion

### 4.1 Climate Model Performance Over the Amazon: Individual and Ensemble Assessment

In this study, we assessed the performance of an ESM ensemble in simulating the onset, end, and length of dry season throughout the Amazon from 2000 to 2014. This was followed by a regional and spatial quantification of future water balance projections by the performance‐weighted ESM ensemble. Our findings show that the ensemble weighting approach generally improves the spatial and temporal representation of the dry season compared to individual CMIP6 ESMs, reducing systematic biases and inter‐model variability. These improvements are consistent with previous studies showing that ensemble‐based approaches enhance climate projection reliability by reducing errors at the global scale (Brunner et al. 2019; Eyring et al. 2019; Castaneda‐Gonzalez et al. 2023). The observed water balance also shows large interannual variability, sometimes exceeding that of the model ensemble. This large spread indicates that the magnitude of the water balance remains uncertain, primarily due to differences in the parameterization of observational evapotranspiration products, while precipitation estimates are comparatively consistent by the use of CHIRPS. Importantly, precipitation estimates across the Amazon can vary substantially among available datasets due to differences in observational inputs and retrieval algorithms (Sapucci et al. 2022), often leading to inconsistencies in the magnitude and direction of trends when compared to station data (Cattelan et al. 2025). The use of a single precipitation dataset may therefore introduce uncertainty in the reference water balance and influence the evaluation and weighting of CMIP6 models. However, as our main results rely on CMIP6 projections, this limitation primarily affects the baseline representation rather than altering the projected future changes. Consequently, model evaluation should be interpreted with caution, as it is constrained by the quality, consistency, and structural assumptions embedded within the reference datasets. Although evaluating individual evapotranspiration products is beyond the scope of this study, the choice of datasets included in the observational ensemble can influence the reference climatology and, consequently, the weighting of CMIP6 models.

Moreover, when models disagree on the sign of precipitation change due to differing circulation responses (Shepherd 2014), opposing signals may be averaged out in a weighted ensemble based on historical performance, reducing its ability to represent future changes. In such cases, alternative ensemble evaluation frameworks, particularly the so‐called storyline approaches, can be applied to decompose ensemble spread in terms of circulation drivers and explore multiple plausible futures (Zappa and Shepherd 2017). However, storyline methods have so far been applied primarily to fixed seasonal changes and have not been extended to assess variations in dry season length. In the Amazon basin, all models project a drying of the southern wet season (Cardoso et al., *in press*) but still exhibit large disagreements in the magnitude of this change. To our knowledge, model disagreement in the dry season length remains to be explored. Hence, the climate‐weighted ESM ensembles remain the most robust and established approach in this context.

The spatial and temporal patterns of dry season dynamics identified here align with those reported by Carvalho et al. (2021), whose framework informed our methodology. While earlier approaches often relied on simplified thresholds to define dry season length, such as constant precipitation cut‐offs or fixed evapotranspiration values (Malhi et al. 2009; Carvalho et al. 2021), these methods may overlook the complexity of drought stress under changing climatic conditions. Our approach advances on constant‐threshold methods by allowing evapotranspiration to vary spatially and temporally. Previous assessments demonstrate that fixed thresholds (e.g., 100 mm/month) can distort estimates of drought extent and severity (Papastefanou et al. 2022), whereas variable evapotranspiration provides a more robust representation of climatic drivers of water stress, including seasonal variation in radiation and vapor pressure deficit (Sun et al. 2019; Baker et al. 2021).

### 4.2 Future Dry Season Projections and Regional Impacts

We find a significant intensification of negative water balance conditions and spatial reorganisation of dry season dynamics across the Amazon by late‐century. While the most pronounced and spatial changes emerge by the end of the century, our projections indicate that drying tendencies are already detectable by mid‐century, suggesting that important shifts in Amazon hydroclimatic seasonality may affect the basin within the next few decades. These earlier signals are particularly concerning because they indicate that the transition toward longer and more intense dry seasons is not solely a late‐century risk, but an ongoing process that will affect ecosystem resilience, fire susceptibility, and regional socioecological systems well before the strongest projected changes materialize.

Regardless of the scenario, the general basin‐wide pattern indicates a drying of the Amazon basin, with the driest period consistently occurring between June and September. However, the spatial patterns reveal strong regional variability, with longer and more severe drought conditions under higher emissions scenarios, particularly under SSP5‐8.5. Seasonal and regional differences are also projected, with dry months becoming drier and transitional months exhibiting marked drying. These shifts, earlier dry season onset and delayed end, are consistent with previous findings linking prolonged dry seasons to climatic warming and further deforestation effects (Brando et al. 2020; Marengo et al. 2018; Fu et al. 2013) as well as altered regional atmospheric circulation (Espinoza et al. 2019; Marengo et al. 2022; Agudelo et al. 2023), reduced moisture transport in northern and southern Amazon (Arias et al. 2023) and the delayed South American Monsoon System (SAMS) (Douville et al. 2021).

The interplay between climate change and land‐use change is central to understanding these drought dynamics. The Amazon may experience increases in temperature up to 3°C under SSP5‐8.5, exacerbating hydrological stress in a region already near the lower rainfall threshold for moist tropical forests (Wainwright et al. 2021). Deforestation amplifies warming effects by reducing evapotranspiration and disrupting moisture recycling (Wright et al. 2017; Leite‐Filho et al. 2019), delaying the wet season onset and prolonging dry periods (Nepstad et al. 2004; O'Connor et al. 2021). However, the magnitude and even the sign of precipitation responses to deforestation remain uncertain, as CMIP6 models show a wide range of outcomes depending on representation of land–atmosphere interactions and circulation feedbacks (Smith, Robertson, et al. 2023). Consistent with this, other studies report increases in drought frequency, length, and intensity across the Amazon, especially in the southern portion (Ukkola et al. 2020; Marengo et al. 2022; Parsons 2020; Eiras‐Barca et al. 2020; Baker et al. 2021; Bottino et al. 2024).

Altered dry season dynamics exert ecological impacts, with cascading effects that extend beyond the Amazon and influence broader South American ecosystems. Longer dry periods reduce soil moisture, constrain tree growth, and increase mortality, particularly among large, slow‐growing trees that store substantial amounts of carbon (Costa et al. 2010; Yao et al. 2024). Moreover, drought intensification raises forest flammability by drying vegetation and reducing atmospheric humidity, therefore creating conditions conducive to more frequent and severe fire events. These conditions align with studies showing that even slight variations in precipitation and temperature can drastically alter fire regimes in the Amazon (Gillett et al. 2004; Tett et al. 2018). Combined with deforestation, land‐use change, and human ignition, fire risk is amplified (Ferreira, Campanharo, Barbosa, et al. 2023; Devisscher et al. 2016; Andela et al. 2017), accelerating forest degradation and potentially transforming these ecosystems from net carbon sinks into carbon sources (Lewis et al. 2011; Gatti et al. 2021; Lapola et al. 2023). This transition could release several petagrams of carbon back into the atmosphere (Malhi et al. 2008; Gatti et al. 2021), reinforcing climate warming through positive feedback loops.

Dry season changes are also connected to large‐scale atmospheric moisture recycling processes, particularly via the South American Monsoon System (SAMS) (Marengo et al. 2012; Londoño Arteaga and Lima 2021). Within this system, the South American Low‐Level Jet (SALLJ) transports moisture from the Amazon toward central and southern regions (Seth et al. 2010; Carvalho et al. 2011). Its intensity and extent are modulated by large scale modes of interannual climate variability, such as the El Niño–Southern Oscillation (ENSO) and the South Atlantic Dipole (SAD), altering the spatial distribution of rainfall across South America. For example, El Niño events enhance moisture transport to Paraguay and southern Brazil, while La Niña suppresses rainfall (Montini et al. 2019). Positive SAD phases lead to low level anticyclonic circulation over the subtropics in the Atlantic during November–January, SALLJ strengthening and the redirection of moisture and augmented precipitation over the SACZ region (Ham et al. 2021) while negative SAD phases are associated with reduced precipitation in the SACZ region. Although some models project future SALLJ intensification (Marengo et al. 2012; Seth et al. 2010; Luo et al. 2022), its capacity to sustain rainfall downstream depends on moisture availability supplied through Amazonian evapotranspiration (Swann and Koven 2017). However, this moisture source is increasingly threatened by intensifying water deficits and widespread forest degradation (Spracklen and Garcia‐Carreras 2015; Yabra et al. 2022). Deforestation reduces evapotranspiration, weakening surface convection and lowering precipitable water by about 7% compared to forested areas (Zemp et al. 2014; Staal et al. 2020; Luo et al. 2022). Observational evidence suggests substantial reductions in precipitation over deforested regions, with decreases of up to 25% reported in tropical regions (Smith, Baker, and Spracklen 2023). This creates a paradox: although SALLJ winds strengthen, reduced moisture supply may limit its effectiveness in sustaining rainfall over central‐southern Brazil, Bolivia, and northern Argentina, particularly during the agricultural growing season (O'Connor et al. 2021; Mu et al. 2023). In addition, delayed wet season onset may exacerbate this feedback, risking a self‐reinforcing cycle of drying with far‐reaching regional and global consequences (Bagley et al. 2014; Brando et al. 2020; Staal et al. 2020; Mu et al. 2023).

Importantly, extension of the dry season also undermines climate mitigation opportunities into interconnected biomes. The Atlantic Forest, one of South America's most fragmented yet biodiversity‐rich regions, retains potential for carbon stocks gains under moderate climate scenarios, if deforestation is curbed (Ferreira, Campanharo, Fonseca, et al. 2023). However, reduced moisture inflow from the Amazon may jeopardize these prospects, amplifying climate stress in regions already vulnerable due to habitat fragmentation (van der Ent et al. 2010, 2014). The Pantanal wetland region, similarly reliant on Amazonian moisture, is highly sensitive to reduced wet season flow, which increases droughts and fire susceptibility (Nobre et al. 2016; Marengo et al. 2021). In fact, the Pantanal has experienced severe droughts and wildfires recently, with over 44,998 km^2^ of native vegetation burned in 2020 alone, approximately 25% of its total area, threatening biodiversity and key ecosystem services such as carbon stocks, fish nursery habitats, and groundwater recharge (Libonati et al. 2022; Shimabukuro et al. 2023).

The socio‐economic implications of the dry season intensification are far‐reaching. Agricultural productivity is highly sensitive to the timing and length of the rainy season (Leite‐Filho et al. 2019, 2021), therefore, longer and drier seasons threaten water availability and disrupt crop calendars in central and southern Brazil, reducing the feasibility of double cropping systems (Cohn et al. 2016). Estimates by Leite‐Filho et al. (2021), under high‐deforestation scenarios, suggest that cumulative losses by 2050 could exceed US$180 billion for beef production and US$5.6 billion for soy, far surpassing the costs of sustainable governance. As precipitation and evapotranspiration patterns are projected to shift, current water transport and irrigation strategies will require reassessment to ensure water management and agricultural resilience (Oliveira et al. 2022). Furthermore, ENSO events may interact with these long‐term changes, amplifying uncertainties in agricultural output (Zemp et al. 2014; Luo et al. 2022). These projected losses provide a strong economic case for strengthening environmental policy and incentivizing low‐deforestation and high climate mitigation pathways. Recent estimates suggest that precipitation from the Amazon forest contributes approximately US$20 ± 7 billion annually to the Brazilian economy (Baker et al. 2026), highlighting the critical economic value of maintaining regional hydrological processes. However, this hydrological service may be increasingly threatened not only by deforestation but also by the physiological effects of rising CO_2_, which can reduce transpiration and moisture recycling, leading to rainfall reductions comparable to large‐scale forest loss (Sampaio et al. 2021).

Mitigation and adaptation strategies must be prioritized to address these challenges. On the mitigation front, reducing greenhouse gas emissions is essential to temper the extreme drying trends, as lower‐emissions scenarios (e.g., SSP126) show considerably less deviation from historical conditions (Ukkola et al. 2020). Strengthening land use planning and enforcing environmental policies are also critical to sustain the hydrological cycle and reduce fire risks (Nepstad et al. 2004; Baker and Spracklen 2022). On the adaptation front, expanding irrigation infrastructure, developing early‐warning systems that integrate short‐term meteorological predictions, implementing strategic fire management, such as Brazil's National Policy on Integrated Fire Management (PNMIF), which promotes controlled burns to reduce late‐season wildfire severity (Carvalho et al. 2021; Silva Junior et al. 2019) could improve preparedness for future drought events, thereby reducing socio‐economic vulnerabilities (Wright et al. 2017).

Beyond national efforts, cross‐border cooperation is essential for addressing shared environmental and climate‐related risks. Regional initiatives such as those led by the Amazon Cooperation Treaty Organization (ACTO) foster coordination, knowledge exchange, and joint development of early‐warning systems and training programs (Amazon Cooperation Treaty Organization (ACTO) 2021). These collaborative efforts also facilitate the alignment of national strategies with regional objectives, helping preserve the ecological integrity of the Amazon and support the livelihoods of its populations under changing climate conditions.

### 4.3 Uncertainties and Limitations

Although the ensemble approach and multi‐model comparisons provided robust insights into dry season dynamics, several uncertainties and limitations remain related to model structure and parameterizations. CMIP6 ESMs vary in the magnitude and spatial distribution of the dry season across the basin due to structural differences, particularly in simulating precipitation regimes and land–atmosphere interactions (Parsons 2020). Systematic biases, including underestimation of precipitation variability, can affect the characterization of dry season onset, end, and length, thereby amplifying uncertainty in hydrological stress estimates (Ukkola et al. 2018). Inter‐model variation in RMSE and the timing of the dry season illustrates the challenge of representing regional‐scale processes. While performance‐based weighting reduces the influence of poorly performing models, it cannot fully correct shared structural biases or incomplete representation of biophysical processes if they exist across the entire ensemble. Consequently, model evaluation should be interpreted with caution, as it is constrained by the quality and consistency of reference datasets. The spatial and seasonal variability of biases suggests that applying bias‐correction techniques or region‐specific weighting strategies may further enhance model performance in representing Amazon drought conditions.

The weighted ensemble tends to overestimate drought conditions in northern and central Amazon from September to March, likely due to biases in precipitation and/or evapotranspiration (Silva et al. 2023). Conversely, dry conditions are generally underestimated from September to December, affecting fire risk assessments, ecological stress monitoring, and hydrological modeling. These spatial divergencies reflect known CMIP6 weaknesses, including underestimation of tropical precipitation linked to misrepresentation of maximum rainfall hot spots and biases in cloud physics, which shift rainfall eastward relative to observations (Firpo et al. 2022; Monteverde et al. 2022). Such limitations align with broader findings of systematic errors in large‐scale circulation and land–atmosphere coupling (Fiedler et al. 2020).

Additional uncertainties arise from intrinsic CMIP6 limitations and datasets resolution. Despite process representation from previous generations (O'Neill et al. 2016; van Vuuren et al. 2017; Tebaldi et al. 2021), CMIP6 models still misrepresent important factors, including some biophysical and vegetation–atmosphere feedback processes and lack of heterogeneity across vegetation types for vegetation–climate interactions, which may lead to conservative projections (Sanderson et al. 2024). In addition, the physiological response of vegetation to rising atmospheric CO_2_ concentrations may influence evapotranspiration and water balance. In CMIP6 ESMs, this process is represented through land surface and vegetation schemes that simulate stomatal conductance responses to CO_2_ (Kooperman et al. 2018; Lawrence et al. 2019; Danabasoglu et al. 2020). Higher CO_2_ concentrations generally reduce stomatal opening, leading to decreased transpiration and increased plant water‐use efficiency (Medlyn et al. 2011; Swann et al. 2016). While this mechanism may partially offset soil moisture depletion at the leaf level, its net effect on evapotranspiration remains uncertain due to interacting physiological responses, vegetation structure, and climatic controls (Vicente‐Serrano et al. 2022). It can also modify land–atmosphere feedbacks by reducing moisture fluxes to the atmosphere and influencing regional precipitation patterns (Lemordant et al. 2018; Kooperman et al. 2018; Sampaio et al. 2021). As our analysis is based on CMIP6 simulations, these processes are implicitly included but cannot be isolated. Therefore, our projected drying reflects the combined influence of CO_2_ physiological responses, warming‐driven increases in atmospheric demand, and changes in precipitation, with the latter likely dominating the overall signal.

Resampling datasets to 1° and monthly time steps enables basin‐scale assessment but limits fine‐scale and short‐term processes detection (Longo et al. 2018). Integrating multiple datasets with differing spatial and temporal resolutions and methodological assumptions can introduce inconsistencies that are difficult to quantify. Therefore, our results should be interpreted as plausible trajectories under specific emission scenarios (e.g., SSP1‐2.6, SSP2‐4.5, and SSP5‐8.5) rather than deterministic predictions. Reducing uncertainties in future work will require expanded observational networks and refined ensemble weighting techniques to strengthen future projections and support planning and adaptation across the Amazon.

## 5 Final Considerations

This study reinforces that the dry season varies spatially and temporally across the basin; thus, assuming a uniform dry season oversimplifies the spatial variability in the Amazon. Here, we provide robust spatially explicit evidence that the Amazon will experience a progressive intensification and lengthening of the dry season under future climate change scenarios. By integrating multi‐source observations with a performance‐weighted CMIP6 ensemble, we show a clear intensification and lengthening of the dry season under all future emission pathways by the end of the century. Even under low‐emission pathways (SSP1‐2.6), one third of the basin will face prolonged dry periods, while under a trajectory broadly consistent with current global emissions trends (SSP2‐4.5), dry season lengthening affects 40% of the basin. These impacts intensify markedly under high‐emission scenarios (SSP5‐8.5), with over half of the Amazon (56.3%) projected to experience at least one additional month of dry conditions by late‐century. These changes reflect a reorganization of regional hydrological dynamics with cascading ecological and socio‐economic consequences.

Furthermore, we show that a performance‐weighted multi‐model ensemble can reduce biases and inter‐model variability, enhancing the accuracy of simulations for water balance and dry season dynamics. These improvements in model performance highlight the value of ensemble‐based approaches for generating more reliable climate projections, especially in data‐sparse and ecologically complex regions like the Amazon. While some spatial biases persist, especially in transitional zones, the results provide insights into the future temporal and spatial distribution of the dry season in the Amazon under moderate to high emission scenarios. The contrast among scenarios illustrates that stronger and earlier emissions reductions could substantially limit dry season intensification, reducing the spatial extent and severity of future hydrological stress.

This finding reinforces the importance of applying scientifically robust and spatially explicit modeling approaches to inform policy decisions. Strengthening environmental governance, implementing climate adaptation plans, and promoting transboundary cooperation are imperative to safeguarding ecological integrity. In doing so, we not only protect a globally critical ecosystem but also contribute to climate mitigation and long‐term resilience of communities dependent on its services.

## Author Contributions

**Igor José Malfetoni Ferreira:** conceptualization, writing – original draft, investigation, methodology, validation, formal analysis, visualization, data curation. **Nathália S. Carvalho:** writing – review and editing, methodology. **Chantelle Burton:** writing – review and editing. **Celso H. L. Silva‐Junior:** writing – review and editing. **Débora Joana Dutra:** writing – review and editing. **Stephen Sitch:** writing – review and editing, resources. **Douglas Kelley:** writing – review and editing. **Luiz E. O. C. Aragão:** writing – review and editing. **Lina M. Mercado:** writing – review and editing, resources. **Dhruba J. Goswami:** writing – review and editing. **Liana O. Anderson:** supervision, writing – review and editing, conceptualization, methodology. **Maria L. F. Barbosa:** writing – review and editing. **Julia Mindlin:** writing – review and editing. **Scott Barningham:** writing – review and editing, resources.

## Funding

This work was supported by Coordenação de Aperfeiçoamento de Pessoal de Nível Superior, Finance Code 001, and 88887.892286/2023‐00. Conselho Nacional de Desenvolvimento Científico e Tecnológico, 381715/2026‐4.

## Conflicts of Interest

The authors declare no conflicts of interest.

## Supporting information


**Figure S1:** Study area resampling to 1° grid cells. Map lines delineate study areas and do not necessarily depict accepted national boundaries.
**Figure S2:** Kruskal‐Wallis test followed by Dunn's post hoc test for significant differences between models.
**Figure S3:** (a) Classification errors of the weighted multimodel ensemble compared to observed data. False positives indicate pixels where the model incorrectly identified a dry region, while false negatives represent pixels where the model failed to detect an observed dry region; n is the total number of misclassified pixels. Total pixel counts (n) is provided for each category (out of 544 total pixels); (b) Spatial biases of the water balance (mm/month) from the weighted multi‐model ensemble, averaged over 2000–2014. Negative values (red shades) indicate an overestimation of drought conditions (drier than observed), while positive values (blue shades) indicate an underestimation (wetter than observed). Map lines delineate study areas and do not necessarily depict accepted national boundaries.
**Figure S4:** Spatial and temporal distribution of the dry season onset, end, and length across the Amazon by the weighted multi‐model ensemble mean for the (a) Historical period, and for (b) mid‐century (2041–2060) under SSP1‐2.6, (c) SSP2‐4.5, and (d) SSP5‐8.5. Map lines delineate study areas and do not necessarily depict accepted national boundaries.
**Figure S5:** Spatial and temporal distribution of the dry season onset, end, and length across the Amazon by the weighted multi‐model ensemble mean for the (a) Historical period, and for (b) late‐century (2081–2100) under SSP1‐2.6, (c) SSP2‐4.5, and (d) SSP5‐8.5. Map lines delineate study areas and do not necessarily depict accepted national boundaries.
**Figure S6:** Spatial changes in the onset, end, and length of the dry season in the Amazon by the weighted multi‐model ensemble mean by mid‐century (2041–2060) under the (a) SSP1‐2.6, (b) SSP2‐4.5, and (c) SSP5‐8.5 climate scenarios compared to the historical period (2000–2014). Positive values indicate a delayed onset or end, and a longer dry season length, while negative values represent an earlier onset or end, and a shorter dry season length. Map lines delineate study areas and do not necessarily depict accepted national boundaries.
**Table S1:** List of the input data used in this study.
**Table S2:** CMIP6 models used in this study.
**Table S3:** Information on CMIP6 Earth System Models used in this study.
**Table S4:** Comparing the observed and weighted multi ensemble dry season (onset and end) for 2000–2014 period.
**Table S5:** Total count of cells for each change category in the onset, end, and length by mid‐century (2041–2060) and late‐century (2081–2100) under SSP126, SSP245, and SSP585 climate scenarios compared to historical period (2000–2014).
**Table S6:** Paired Wilcoxon test for monthly future water balance estimates in under SSP126, SSP245, and SSP585 (late‐century: 2081–2100) climate scenarios compared to historical period (2000–2014), considering the median location shifts greater or less than zero as an alternative hypothesis and at a significance level of 0.05.
**Table S7:** Paired Wilcoxon test for annual mean water balance estimates under SSP126, SSP245, and SSP585 (late‐century: 2081–2100) climate scenarios compared to historical period (2000–2014), considering the median location shifts greater or less than zero as an alternative hypothesis and at a significance level of 0.05.
**Appendix S1:** Remote sensing based data description.
**Appendix S2:** Shared socioeconomic pathways.

## Data Availability

The data that support the findings of this study are openly available in Zenodo at https://doi.org/10.5281/zenodo.18260083.
