# Supplementary material for: Amazon Dry Season Will Lengthen Under Future Climate
Source: Glob Chang Biol. 2026 Jul 23;32(7):e71018. doi: 10.1111/gcb.71018 (PMC13392597; doi:10.1111/gcb.71018)
Supplement: Supplementary file 1 — Figure S1: Study area resampling to 1° grid cells. Map lines delineate study areas and do not necessarily depict accepted national boundaries. Figure S2: Kruskal‐Wallis test followed by Dunn's post hoc test for significant differences between models. Figure S3: (a) Classification errors of the weighted multimodel ensemble compared to observed data. False positives indicate pixels where the model incorrectly identified a dry region, while false negatives represent pixels where the model failed to detect an observed dry region; n is the total number of misclassified pixels. Total pixel counts (n) is provided for each category (out of 544 total pixels); (b) Spatial biases of the water balance (mm/month) from the weighted multi‐model ensemble, averaged over 2000–2014. Negative values (red shades) indicate an overestimation of drought conditions (drier than observed), while positive values (blue shades) indicate an underestimation (wetter than observed). Map lines delineate study areas and do not necessarily depict accepted national boundaries. Figure S4: Spatial and temporal distribution of the dry season onset, end, and length across the Amazon by the weighted multi‐model ensemble mean for the (a) Historical period, and for (b) mid‐century (2041–2060) under SSP1‐2.6, (c) SSP2‐4.5, and (d) SSP5‐8.5. Map lines delineate study areas and do not necessarily depict accepted national boundaries. Figure S5: Spatial and temporal distribution of the dry season onset, end, and length across the Amazon by the weighted multi‐model ensemble mean for the (a) Historical period, and for (b) late‐century (2081–2100) under SSP1‐2.6, (c) SSP2‐4.5, and (d) SSP5‐8.5. Map lines delineate study areas and do not necessarily depict accepted national boundaries. Figure S6: Spatial changes in the onset, end, and length of the dry season in the Amazon by the weighted multi‐model ensemble mean by mid‐century (2041–2060) under the (a) SSP1‐2.6, (b) SSP2‐4.5, and (c) SSP5‐8.5 climate scenarios compar [file GCB-32-e71018-s001.docx]

**Amazon Dry Season Will Lengthen Under Future Climate**

Igor José Malfetoni Ferreira ^1^, Nathália S. Carvalho ^3^, Lina M. Mercado ^4,5^, Stephen Sitch ^4^, Douglas Kelley ^6^, Chantelle Burton ^7^, Débora Joana Dutra ^1^, Scott Barningham ^4^, Maria L. F. Barbosa ^6^, Julia Mindlin ^8^, Celso H. L. Silva-Junior ^9,10^, Dhruba J. Goswami ^4^, Luiz E. O. C. Aragão ^1,2,4^, and Liana O. Anderson^1,2^

^1^ Remote Sensing Postgraduate Program (PGSER), Coordination for Education, Research and Outreach (COEPE), Brazil’s National Institute for Space Research (INPE), São José dos Campos-SP, Brazil

^2^ Earth Observation and Geoinformatics Division (DIOTG), Earth Sciences General Coordination (CGCT), Brazil’s National Institute for Space Research (INPE), São José dos Campos-SP, Brazil

^3^ Lancaster Environment Centre, Lancaster University, Lancaster, Lancashire, LA1 4YQ, United Kingdom

^4^ Faculty of Environment, Science and Economy, University of Exeter, United Kingdom

^5^ Land and Climate Science, UK Centre for Ecology & Hydrology, Wallingford, United Kingdom

^6^ Water and Climate Science, UK Centre for Ecology & Hydrology, Wallingford, United Kingdom

^7^ Met Office Hadley Centre, Exeter EX1 3PB, United Kingdom

^8^ Leipzig Institute for Meteorology, Leipzig University, Leipzig, 04103, Germany

^9^ Amazon Environmental Research Institute (IPAM), Brasília- DF, 70863-520, Brazil

^10^ Federal University of Maranhão (UFMA), São Luís-MA, 65085-580, Brazil

**Supporting Information Tables**

**Table S1**. List of the input data used in this study

| Product | Spatial Res. | Description | Source |
| --- | --- | --- | --- |
| MODIS/ MOD16A2GF | 0.05° | Remote sensing-based evapotranspiration product (MODIS), derived from land surface temperature, humidity, solar radiation, and vegetation indices. | NASA Goddard Space Flight Center  (Mu et al. 2011a) |
| GLDAS | 0.25° | Data assimilation system that integrates observations and modelling to estimate land and atmospheric variables, including evapotranspiration. | NASA Goddard Space Flight Center  (Rodell et al., 2004) |
| FLDAS | 0.1° | Similar to GLDAS but optimized for specific regions, focusing on food security by incorporating satellite and observational data. | Famine Early Warning Systems Network  (McNally et al., 2017) |
| GLEAM | 0.25° | Satellite-based ET estimates derived from solar radiation and soil moisture data, without reanalysis assimilation. | Vrije Universiteit Amsterdam  (Martens et al. 2017) |
| AET/ TerraClimate | 0.04° | Provides actual evapotranspiration (AET) based on a combination of satellite data, Thornthwaite-Mather water balance, and Penman-Monteith equation, considering actual water availability. | University of Idaho  (Abatzoglou et al. 2018) |
| CHIRPS | 0.05° | Satellite-derived precipitation estimates combined with global rain gauge records. | Climate Hazards Center, University of California  (Funk et al., 2015) |

**Table S2**. CMIP6 models used in this study.

| **Model** | **Members** | **Institution** | **Reference** |
| --- | --- | --- | --- |
| BCC-CSM2-MR | r1i1f1p1 | Beijing Climate Center, China Meteorological Administration, China | Xin et al., (2018) |
| CESM2 | r4i1f1p1, r10i1f1p1, r11i1f1p1 | National Center for Atmospheric Research (NCAR), EUA | Danabasoglu, (2019) |
| CESM2-WACCM | r1i1f1p1 | National Center for Atmospheric Research (NCAR), EUA | Danabasoglu, (2019) |
| CMCC-ESM2 | r1i1f1p1 | Centro Euro-Mediterraneo sui Cambiamenti Climatici, Italy | Lovato & Peano, (2021) |
| FIO-ESM2-0 | r1i1f1p1, r2i1f1p1, r3i1f1p1 | First Institute of Oceanography, China | Song et al., (2019) |
| GFDL-ESM4 | r1i1f1p1 | NOAA Geophysical Fluid Dynamics Laboratory (GFDL), EUA | Dunne et al., (2020) |
| MPI-ESM1-2-HR | r1i1f1p1, r2i1f1p1 | Max Planck Institute for Meteorology, Germany | Wieners et al., (2022) |
| MRI-ESM2-0 | r1i1f1p1, r2i1f1p1, r3i1f1p1, r4i1f1p1, r5i1f1p1 | Meteorological Research Institute, Meteorological Agency, Japan | Yukimoto et al., (2019) |
| NorESM2-MM | r1i1f1p1 | Norwegian Meteorological Institute, Norway | Bentsen et al., (2022) |
| TaiESM1 | r1i1f1p1 | Research Center for Environmental Changes, Taiwan | Lee & Liang, (2022) |

**Table S3.** Information on CMIP6 Earth System Models used in this study.

| **Model** | **Parameterization / Spatial Res.** | **Advantages** | **Disadvantages** | **Usefulness for Amazon Region** | **Reference** |
| --- | --- | --- | --- | --- | --- |
| BCC-CSM2-MR | Coupled model focusing on long-term climate projections and interactions between atmosphere, ocean, and biogeophysics  1.125°, 1.121277° | Strong performance in simulating global climatic events and tropical precipitation patterns | Spatial resolution may not be sufficient for detailed regional modelling | Suitable for global projections but not for detailed Amazon analysis | Xin et al. (2018) |
| CESM2 | Coupled model with a strong focus on atmosphere-ocean interactions and biogeophysical processes.  1.25°, 0.9424084° | High capability to simulate extreme climate events and precipitation patterns | Spatial resolution may not be ideal for capturing local variations in tropical regions | Useful for extreme event studies, but limited to detailed Amazon regional analysis | Danabasoglu, (2019) |
| CESM2-WACCM | High-top atmospheric model extending into the mesosphere, focusing on atmospheric chemistry and dynamics.  1.25°, 0.9424084° | Enhanced representation of atmospheric chemistry and stratospheric processes | Increased complexity and computational demand; spatial resolution may limit regional applicability | Useful for studying atmospheric chemistry impacts on Amazon climate, but spatial resolution may limit detailed regional analysis | Danabasoglu (2019) |
| CMCC-ESM2 | Coupled model focusing on global climatic variations and atmospheric interactions.  1.125°, 1.121277° | Well-suited for simulating large-scale climatic phenomena and long-term projections | Spatial resolution may be insufficient for regional modeling. | Provides general insights but not ideal for precise regional projections of the Amazon | Lovato & Peano, (2021) |
| FIO-ESM2-0 | Coupled model integrating ocean-atmosphere interactions with a focus on ocean dynamics.  1.25°, 0.9424084° | Effective in simulating oceanic processes and their climatic impacts | Limited validation over the Amazon region; spatial resolution may be coarse | Potentially useful for studies involving oceanic influences on Amazon climate, but requires further validation | Song et al., (2019) |
| GFDL-ESM4 | Coupled model with an emphasis on atmospheric, oceanic, and biogeophysical processes  1.25°, 1.0° | Capable of simulating global climatic events and droughts | Spatial resolution limits capturing local climatic variability | Suitable for simulating global phenomena, but limited to local Amazon analysis | Krasting et al. (2018) |
| MPI-ESM1-2-HR | Coupled model focusing on long-term climate simulations and climatic variability.  0.9375°, 0.9349134° | Suitable for simulating long-term climate change and temperature variations | Spatial resolution may not be ideal for tropical regions with high climatic diversity | Suitable for global climate trends but limited to Amazon simulations. | Jungclaus et al. (2019) |
| MRI-ESM2-0 | Coupled model with a focus on global and regional climate changes  1.125°, 1.121277° | Strong in simulating precipitation patterns and global climatic variability | Spatial resolution may not be ideal for capturing local climatic variability | Useful for global climate trends but not detailed Amazon regional analysis | Yukimoto et al. (2019) |
| NorESM2-MM | Coupled model with interactions between climatic and oceanic processes  1.25°, 0.9424084° | Strong performance in long-term climate simulations. | Spatial resolution may not be ideal for detailed Amazon simulations | Useful for global climate analysis but limited to detailed Amazon simulations | Bentsen et al. (2019) |
| TaiESM1 | Coupled model with a strong focus on atmospheric and oceanic processes  1.25°, 0.9424084° | Suitable for simulating prolonged droughts and tropical climate changes | Limited spatial resolution for detailed Amazon simulations | Useful for global climate patterns, but limited to Amazon-specific modeling. | Lee and Liang (2020) |

**Table S4**. Comparing the observed and weighted multi ensemble dry season (onset and end) for 2000-2014 period

| Observed Dry Season | | | | | |
| --- | --- | --- | --- | --- | --- |
| Onset | | End | | Length | |
| Month | Area (%) | Month | Area (%) | Number of Months | Area (%) |
| No dry season | 13.20 | No dry season | 13.20 | No dry season | 13.20 |
| Jan | 4.23 | Jan | 1.10 | 1 | 4.23 |
| Feb | 2.21 | Feb | 4.23 | 2 | 7.17 |
| Mar | 0.73 | Mar | 5.15 | 3 | 12.50 |
| Apr | 5.51 | Apr | 2.94 | 4 | 27.00 |
| May | 22.10 | May | 0 | 5 | 24.68 |
| Jun | 19.30 | Jun | 0 | 6 | 6.07 |
| Jul | 9.74 | Jul | 0 | 7 | 3.49 |
| Aug | 12.90 | Aug | 15.30 | 8 | 1.29 |
| Sep | 5.51 | Sep | 34.90 | 9 | 0.37 |
| Oct | 1.29 | Oct | 7.35 | 10 | 0 |
| Nov | 0.37 | Nov | 13.26 | 11 | 0 |
| Dec | 2.91 | Dec | 2.57 | 12 | 0 |
| Weighted Multimodel Ensemble Dry Season | | | | | |
| Onset | | End | | Length | |
| Month | Area (%) | Month | Area (%) | Number of Months | Area (%) |
| No dry season | 2.02 | No dry season | 2.02 | No dry season | 2.02 |
| Jan | 6.25 | Jan | 1.10 | 1 | 2.39 |
| Feb | 0 | Feb | 6.25 | 2 | 8.46 |
| Mar | 0 | Mar | 9.56 | 3 | 11.20 |
| Apr | 2.92 | Apr | 5.15 | 4 | 22.60 |
| May | 27.40 | May | 0 | 5 | 28.32 |
| Jun | 20.60 | Jun | 0 | 6 | 17.30 |
| Jul | 22.80 | Jul | 0.19 | 7 | 3.31 |
| Aug | 3.31 | Aug | 8.46 | 8 | 3.68 |
| Sep | 6.25 | Sep | 36.20 | 9 | 0.55 |
| Oct | 1.65 | Oct | 22.80 | 10 | 0.18 |
| Nov | 1.65 | Nov | 7.35 | 11 | 0 |
| Dec | 5.15 | Dec | 0.92 | 12 | 0 |

**Table S5.** Total count of cells for each change category in the onset, end, and length by mid-century (2041–2060) and late-century (2081–2100) under SSP126, SSP245, and SSP585 climate scenarios compared to historical period (2000-2014).

| Mid-century (2041-2060) | | | | | | | Late-century (2081-2100) | | | | | |
| --- | --- | --- | --- | --- | --- | --- | --- | --- | --- | --- | --- | --- |
|  | Onset | | End | | Length | | Onset | | End | | Length | |
|  | SSP126 | | | | | | SSP126 | | | | | |
| Change | Count | (%) | Count | (%) | Count | (%) | Count | (%) | Count | (%) | Count | (%) |
| < -2 | 1 | 0,2 | 6 | 1,1 | 2 | 0,4 | 4 | 0,8 | 8 | 1,5 | 2 | 0,4 |
| -2 | 0 | 0 | 0 | 0 | 3 | 0,6 | 1 | 0,2 | 0 | 0 | 4 | 0,7 |
| -1 | 95 | 17,8 | 7 | 1,3 | 12 | 2,2 | 120 | 22,5 | 17 | 3,2 | 16 | 2,9 |
| **0** | **415** | **77,6** | **417** | **77,9** | **341** | **62,7** | **393** | **73,7** | **418** | **78,4** | **330** | **60,7** |
| 1 | 12 | 2,2 | 101 | 18,9 | 164 | 30,1 | 8 | 1,5 | 87 | 16,3 | 170 | 31,3 |
| 2 | 3 | 0,6 | 0 | 0 | 16 | 2,9 | 1 | 0,2 | 0 | 0 | 19 | 3,5 |
| > 2 | 9 | 1,7 | 4 | 0,7 | 6 | 1,1 | 6 | 1,1 | 3 | 0,6 | 3 | 0,6 |
|  | SSP245 | | | | | | SSP245 | | | | | |
| Change | Count | (%) | Count | (%) | Count | (%) | Count | (%) | Count | (%) | Count | (%) |
| < -2 | 3 | 0,6 | 10 | 1,9 | 4 | 0,7 | 7 | 1,3 | 8 | 1,5 | 1 | 0,2 |
| -2 | 0 | 0 | 0 | 0 | 0 | 0 | 2 | 0,4 | 0 | 0 | 5 | 0,9 |
| -1 | 81 | 15,2 | 17 | 3,2 | 25 | 4,6 | 67 | 12,5 | 24 | 4,5 | 31 | 5,7 |
| **0** | **429** | **80,3** | **402** | **75,3** | **341** | **62,7** | **433** | **81,1** | **323** | **60,5** | **285** | **52,4** |
| 1 | 12 | 2,2 | 101 | 18,9 | 155 | 28,5 | 17 | 3,2 | 172 | 32,2 | 187 | 34,4 |
| 2 | 0 | 0 | 0 | 0 | 15 | 2,8 | 5 | 0,9 | 2 | 0,4 | 29 | 5,3 |
| > 2 | 9 | 1,7 | 4 | 0,7 | 4 | 0,7 | 3 | 0,6 | 5 | 0,9 | 6 | 1,1 |
|  | SSP585 | | | | | | SSP585 | | | | | |
| Change | Count | (%) | Count | (%) | Count | (%) | Count | (%) | Count | (%) | Count | (%) |
| < -2 | 2 | 0,4 | 9 | 1,7 | 5 | 0,9 | 15 | 2,8 | 14 | 2,6 | 1 | 0,2 |
| -2 | 0 | 0 | 0 | 0 | 4 | 0,7 | 2 | 0,4 | 0 | 0 | 1 | 0,2 |
| -1 | 114 | 21,3 | 33 | 6,2 | 29 | 5,3 | 95 | 17,7 | 15 | 2,8 | 16 | 2,9 |
| **0** | **396** | **73,9** | **374** | **69,8** | **294** | **54,0** | **398** | **74,1** | **277** | **51,6** | **220** | **40,4** |
| 1 | 7 | 1,3 | 114 | 21,3 | 180 | 33,1 | 18 | 3,4 | 215 | 40,0 | 255 | 46,9 |
| 2 | 0 | 0 | 0 | 0 | 25 | 4,6 | 2 | 0,4 | 9 | 1,7 | 37 | 6,8 |
| > 2 | 17 | 3,2 | 6 | 1,1 | 7 | 1,3 | 7 | 1,3 | 7 | 1,3 | 14 | 2,6 |

**Table S6.** Paired Wilcoxon test for monthly future water balance estimates in under SSP126, SSP245, and SSP585 (late-century: 2081-2100) climate scenarios compared to historical period (2000-2014), considering the median location shifts greater or less than zero as an alternative hypothesis and at a significance level of 0.05.

| Scenario | Month | W statistic | p-value | Historical Water Balance Median (mm/month) | Future Water Balance Median (mm/month) |
| --- | --- | --- | --- | --- | --- |
|  |  |  |  |  |  |
|  |  |  |  |  |  |
| SSP126 | Jan | 75550 | 1,38E-05 | 115,0 | 120,4 |
|  | Feb | 85766 | 4,25E-02 | 118,4 | 117,3 |
|  | Mar | 148633 | 2,08E-34 | 141,2 | 130,0 |
|  | Apr | 119224 | 2,68E-08 | 113,6 | 105,8 |
|  | May | 159127 | 2,31E-48 | 43,4 | 26,8 |
|  | Jun | 122594 | 2,52E-10 | -27,6 | -34,3 |
|  | Jul | 104653 | 2,41E-02 | -53,3 | -51,4 |
|  | Aug | 129576 | 2,58E-15 | -59,0 | -59,8 |
|  | Sep | 131112 | 1,48E-16 | -36,0 | -40,9 |
|  | Oct | 177861 | 2,29E-79 | 13,4 | -2,7 |
|  | Nov | 135371 | 2,86E-20 | 92,5 | 82,5 |
|  | Dec | 84249 | 1,76E-02 | 124,5 | 123,4 |
| SSP245 | Jan | 64042 | 3,47E-12 | 115,0 | 129,7 |
|  | Feb | 133515 | 1,33E-18 | 118,4 | 109,4 |
|  | Mar | 159891 | 1,79E-49 | 141,2 | 122,5 |
|  | Apr | 137998 | 9,22E-23 | 113,6 | 101,1 |
|  | May | 157348 | 7,97E-46 | 43,4 | 22,9 |
|  | Jun | 89532 | 2,40E-01 | -27,6 | -26,8 |
|  | Jul | 72352 | 3,94E-07 | -53,3 | -49,4 |
|  | Aug | 81498 | 2,72E-03 | -59,0 | -55,2 |
|  | Sep | 125268 | 4,14E-12 | -36,0 | -40,8 |
|  | Oct | 180499 | 2,32E-84 | 13,4 | -19,8 |
|  | Nov | 180245 | 7,13E-84 | 92,5 | 59,2 |
|  | Dec | 92962 | 6,92E-01 | 124,5 | 121,8 |
| SSP585 | Jan | 141711 | 1,53E-26 | 115,0 | 99,9 |
|  | Feb | 146774 | 3,43E-32 | 118,4 | 82,5 |
|  | Mar | 170960 | 4,89E-67 | 141,2 | 105,3 |
|  | Apr | 174440 | 4,02E-73 | 113,6 | 89,8 |
|  | May | 151268 | 1,11E-37 | 43,4 | 24,8 |
|  | Jun | 117927 | 1,39E-07 | -27,6 | -32,1 |
|  | Jul | 73985 | 2,58E-06 | -53,3 | -47,8 |
|  | Aug | 93339 | 7,56E-01 | -59,0 | -55,6 |
|  | Sep | 129151 | 5,58E-15 | -36,0 | -41,5 |
|  | Oct | 182255 | 9,02E-88 | 13,4 | -29,5 |
|  | Nov | 181548 | 2,17E-86 | 92,5 | 42,2 |
|  | Dec | 85102 | 2,93E-02 | 124,5 | 127,0 |

**Table S7.** Paired Wilcoxon test for annual mean water balance estimates under SSP126, SSP245, and SSP585 (late-century: 2081-2100) climate scenarios compared to historical period (2000-2014), considering the median location shifts greater or less than zero as an alternative hypothesis and at a significance level of 0.05.

| Scenario | Historical Water Balance Median (mm/month) | Future Water Balance Median (mm/month) | W statistic | p-value |
| --- | --- | --- | --- | --- |
|  |  |  |  |  |
|  |  |  |  |  |
| SSP126 | 55,0 | 50,0 | 179712 | 7,44E-83 |
| SSP245 | 55,0 | 44,6 | 185399 | 4,76E-94 |
| SSP585 | 55,0 | 39,2 | 188470 | 2,16 E-90 |

**Supporting Information Figures**

**
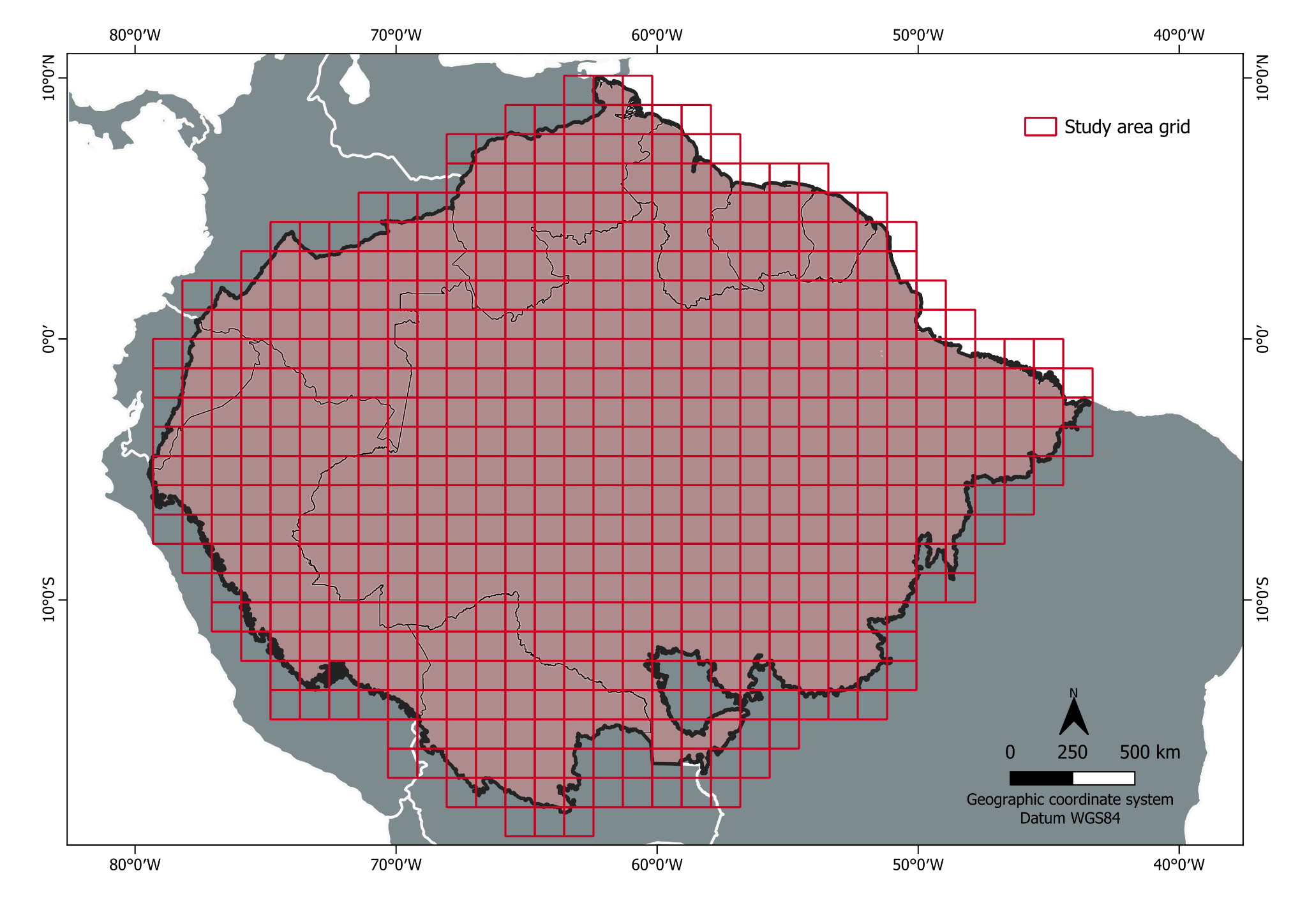
**

**Figure S1.** Study area resampling to 1° grid cells. Map lines delineate study areas and do not necessarily depict accepted national boundaries.


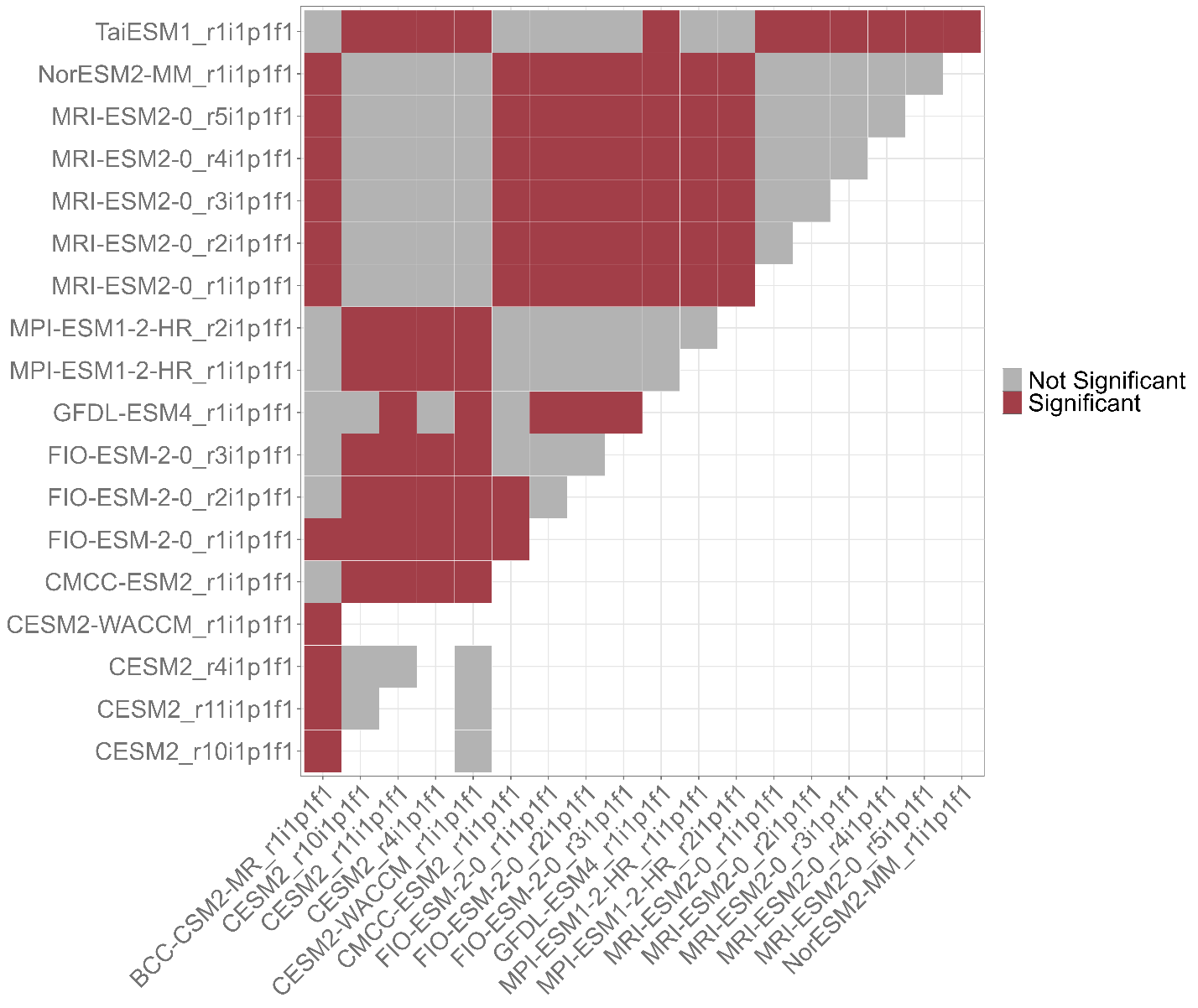


**Figure S2.** Kruskal-Wallis test followed by Dunn’s post hoc test for significant differences between models.

**Spatial variability of water balance:** Spatial evaluation demonstrates that the weighted multimodel ensemble effectively captures the spatial dynamics of the dry season throughout the year (Figure 3a), although classification errors are more common in transition zones, especially as the drought conditions expand. This indicates that model uncertainties are primarily associated with the spatial delineation of seasonal drying than the overall detection. False positives are more frequent from July to October, peaking in September (n = 116) but also increase from December to February. False negatives are predominantly observed from September to November, peaking in November (n = 41). Strong biases occur in the northeast Amazon from January to April (±150 mm/month), with further negative biases in July (northwest) and positive biases in December (southwest), as shown in Figure 3b. These patterns suggest a model’s tendency to overestimate the drought extension during the early dry season, when more than 50% of the Amazon experiences dry conditions, and underestimate it during the wet season transition.


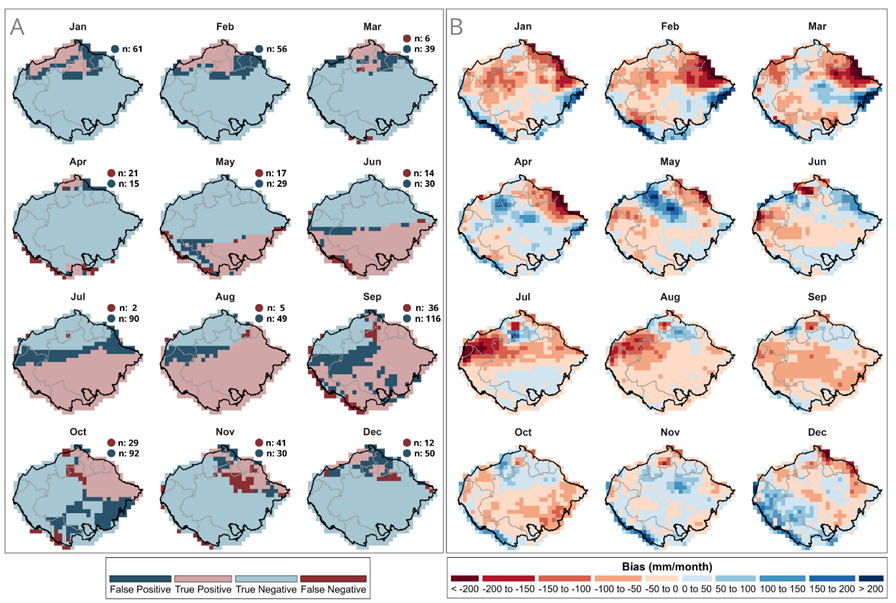


**Figure S3.** (a) Classification errors of the weighted multimodel ensemble compared to observed data. False positives indicate pixels where the model incorrectly identified a dry region, while false negatives represent pixels where the model failed to detect an observed dry region; n is the total number of misclassified pixels. Total pixel counts (n) is provided for each category (out of 544 total pixels); (b) Spatial biases of the water balance (mm/month) from the weighted multi-model ensemble, averaged over 2000-2014. Negative values (red shades) indicate an overestimation of drought conditions (drier than observed), while positive values (blue shades) indicate an underestimation (wetter than observed). Map lines delineate study areas and do not necessarily depict accepted national boundaries.


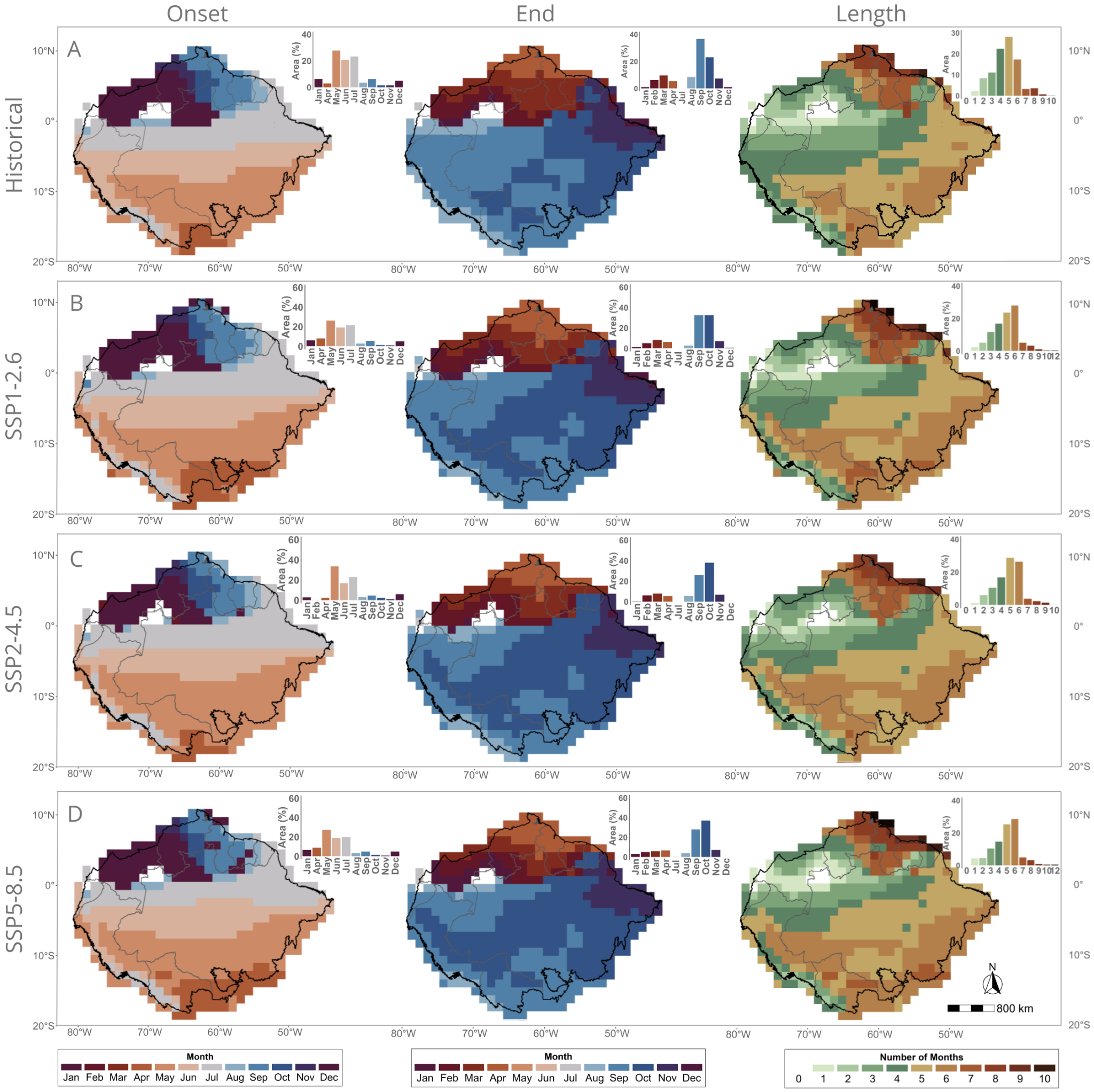


**Figure S4.** Spatial and temporal distribution of the dry season onset, end, and length across the Amazon by the weighted multi-model ensemble mean for the (a) Historical period, and for (b) mid-century (2041–2060) under SSP1-2.6, (c) SSP2-4.5, and (d) SSP5-8.5. Map lines delineate study areas and do not necessarily depict accepted national boundaries.


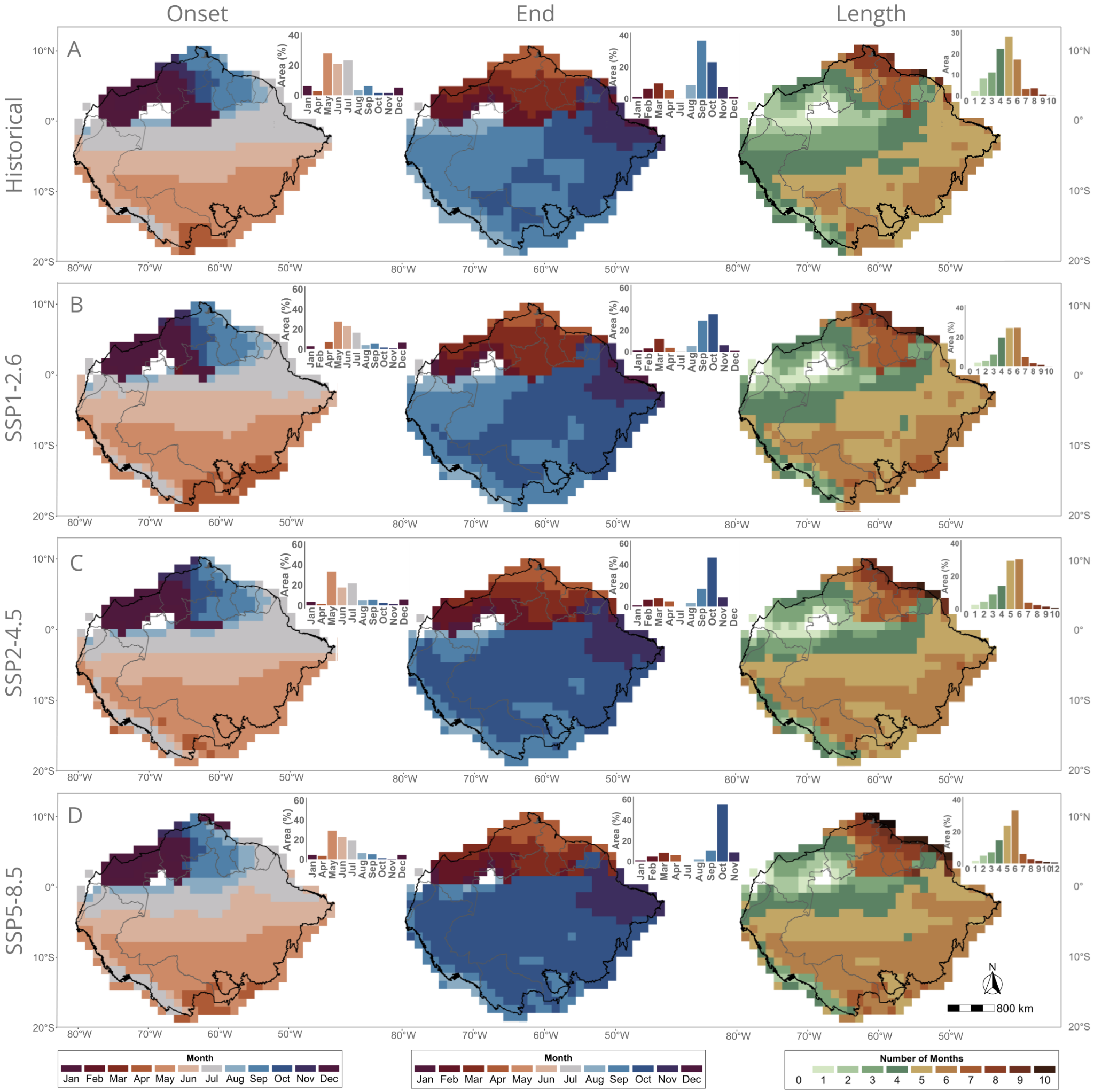


**Figure S5**. Spatial and temporal distribution of the dry season onset, end, and length across the Amazon by the weighted multi-model ensemble mean for the (a) Historical period, and for (b) late-century (2081-2100) under SSP1-2.6, (c) SSP2-4.5, and (d) SSP5-8.5. Map lines delineate study areas and do not necessarily depict accepted national boundaries.


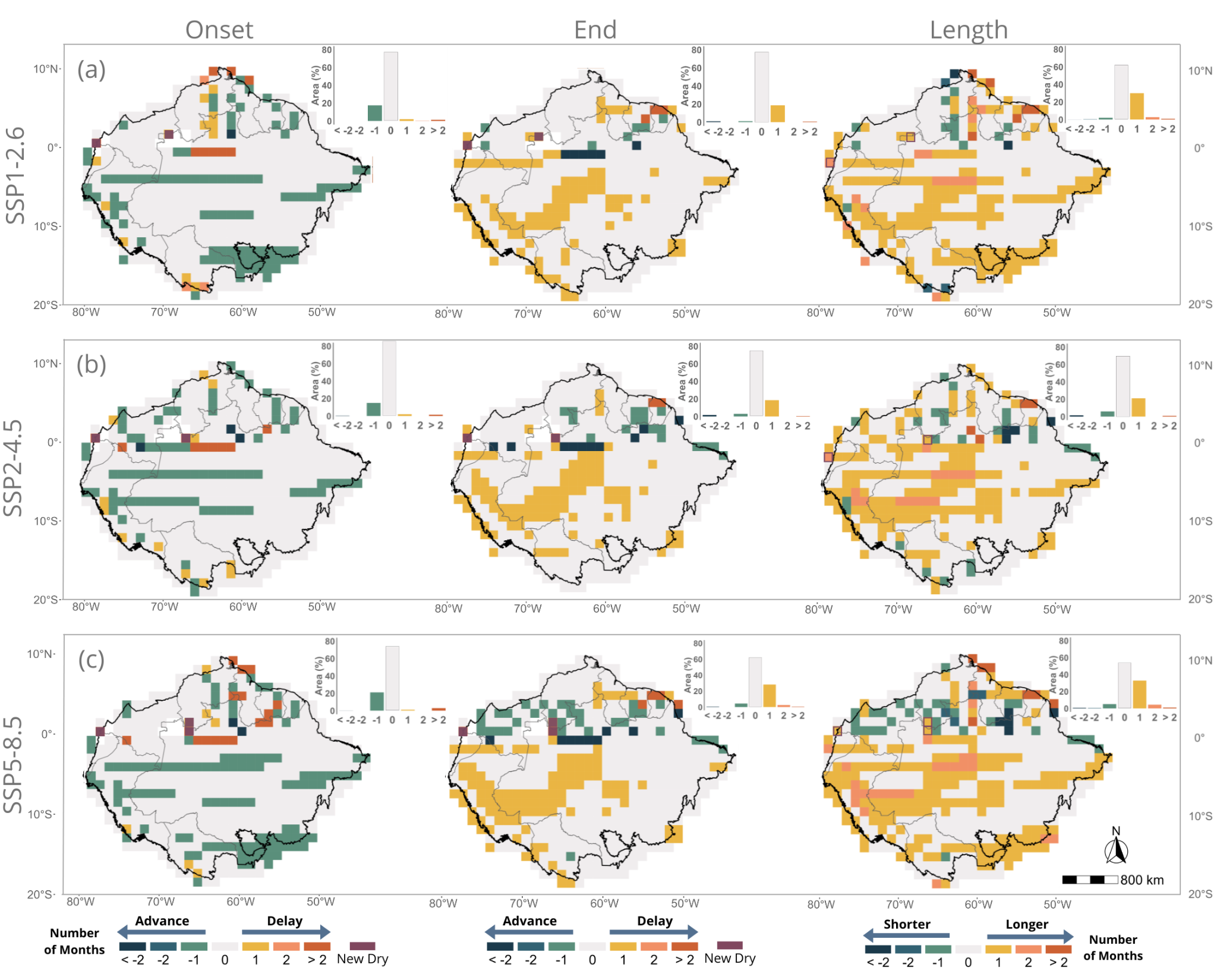
**Figure S6.** Spatial changes in the onset, end, and length of the dry season in the Amazon by the weighted multi-model ensemble mean by mid-century (2041–2060) under the (a) SSP1-2.6, (b) SSP2-4.5, and (c) SSP5-8.5 climate scenarios compared to the historical period (2000-2014). Positive values indicate a delayed onset or end, and a longer dry season length, while negative values represent an earlier onset or end, and a shorter dry season length. Map lines delineate study areas and do not necessarily depict accepted national boundaries.

**Supporting Information Appendix S1: Remote sensing based data description**

In this study, we used precipitation data alongside evapotranspiration estimates to assess water stress in forests across the Amazon Basin (Table S1). Precipitation data were combined with five different evapotranspiration models to represent observed or ground-truth conditions, serving as a baseline for subsequent comparisons with modelled outputs.

Monthly precipitation data were obtained from the Climate Hazards Group InfraRed Precipitation with Station (CHIRPS) platform (https://www.chc.ucsb.edu/data/chirps), which integrates satellite observations, precipitation estimates, and ground station into a spatially and temporally consistent grid suitable for trend analysis (Funk et al., 2015). CHIRPS has been validated globally (Duan et al., 2016; Katsanos et al., 2016; Perdigon-Morales et al., 2018), including in the Amazon, where it explains 73% of the variance in rain gauge data (Anderson et al. 2018), confirming its reliability for large-scale hydrological studies In the Amazon.

For evapotranspiration, we utilized multiple satellite based data and reanalysis products to enhance robustness. The MOD16A2GF is global evapotranspiration product that provides 8-day actual evapotranspiration estimates at 500 m resolution using ground-based meteorological observations and remote sensing data from the Moderate Resolution Imaging Spectroradiometer (MODIS) sensor (Mu et al., 2011a,b). GLDAS and FLDAS, developed by NASA, integrate satellite data and ground-based observations to estimate land surface and water fluxes (Rodell et al., 2004; McNally et al., 2017). GLEAM product estimates land evaporation and soil moisture using satellite data (Miralles et al., 2011). Finally, TerraClimate offers monthly climate and water balance variables at high resolution using the Penman-Monteith equation (Abatzoglou et al., 2018).

**Supporting Information Appendix S2: Shared Socioeconomic Pathways**

In the sixth phase of the Coupled Model Intercomparison Project (CMIP6), Earth system models exhibit enhanced spatial and temporal resolutions for both atmosphere and ocean components, alongside more sophisticated physical parameterizations, most notably an improved representation of aerosols and their interactions with clouds, compared to CMIP5 models (Wyser et al., 2019; Collazo et al., 2022).To coordinate future‐projection experiments across the climate, integrated assessment, and impacts communities, CMIP6 established the Scenario Model Intercomparison Project (ScenarioMIP), which provides consistent, spatially explicit emissions and land‐use forcings and defines standardized experiments based on alternative future trajectories (O’Neil et al., 2016; IPCC, 2022).

Under the ScenarioMIP, the Shared Socioeconomic Pathways (SSPs) define five qualitatively distinct narratives of global societal development, each quantified in terms of demographic, economic, technological, and policy-relevant framework to span a wide range of challenges to climate change mitigation and adaptation (KC & Lutz, 2017; Jiang and O’Neill, 2017; Dellink et al., 2017; O’Neil et al., 2017). SSP1 (“Sustainability”) describes a world of inclusive, green growth with low resource intensity (van Vuuren et al.,2017); SSP2 (“Middle of the Road”) follows historical social and economic trends, presenting moderate challenges (Fricko et al.,2017); SSP3 (“Regional Rivalry”) emphasizes nationalist policies and fragmented development, resulting in high vulnerabilities (Fujimori et al., 2017); SSP4 (“Inequality”) portrays deepening disparities within and between nations (Calvin et al., 2017); and SSP5 (“Fossil‑Fueled Development”) assumes rapid economic growth powered by abundant fossil resources (Kriegler et al., 2017). By developing climate and societal futures, the new framework combines the SSPs and Representative Concentration Pathways (RCPs), from CMIP5 framework, in a Scenario Matrix Architecture, linking socioeconomic storylines with radiative forcing pathways, thereby enabling integrated assessments of impacts, adaptation, and mitigation (Riahi et al., 2017; O’Neil et al., 2020).

The four RCPs (RCP2.6, RCP4.5, RCP6.0, and RCP8.5) are named after the level of radiative forcing they project by the year 2100, measured in watts per square meter (W/m²) (Moss et al., 2010). RCP2.6 represents a stringent mitigation trajectory peaking near 3 W m⁻² before declining to 2.6 W m⁻² (Van Vuuren et al., 2007; Van Vuuren et al., 2011); RCP4.5 and RCP6.0 stabilize at their respective forcing levels after 2100 (Thomson et al., 2011; Masui et al, 2011); and RCP8.5 continues rising throughout the century, peaking 8.5 W m⁻² by 2100 (Riahi et al., 2011). The integration of SSPs allows a systematic examination of climate outcomes based on varying socioeconomic developments and emission trajectories. For instance, the SSP1-2.6 combination represents a sustainable world with strong climate policies, aiming to limit global warming to approximately 1.8°C by 2100. SSP2-4.5, follow historical trends with moderate mitigation challenges, resulting in an estimated warming of around 2.7°C. Conversely, the SSP5-8.5 scenario depicts a fossil-fuelled development path with minimal mitigation efforts, potentially leading to warming exceeding 4°C by the end of the century (Riahi et al., 2017; O’Neill et al., 2017).

In summary, the SSP-RCP framework provides a robust tool for exploring the interplay between socioeconomic pathways and climate outcomes. By considering a range of narratives and emission trajectories, researchers and policymakers can better understand potential futures and devise strategies to address the multifaceted challenges posed by climate change.

**References**

Abatzoglou, J. T., Dobrowski, S. Z., Parks, S. A., & Hegewisch, K. C. (2018). TerraClimate, a high-resolution global dataset of monthly climate and climatic water balance from 1958–2015. Scientific Data, 5(1), 170191. https://doi.org/10.1038/sdata.2017.191

Anderson, L. O., Neto, G. R., Cunha, A. P., Fonseca, M. G., de Moura, Y. M., Dalagnol, R., Wagner, F. H., & de Aragão, L. E. O. E. C. (2018). Vulnerability of Amazonian forests to repeated droughts. Philosophical Transactions of the Royal Society B: Biological Sciences, 373(1760). https://doi.org/10.1098/rstb.2017.0411

Bentsen, M., Oliviè, D. J. L., Seland, O., Toniazzo, T., Gjermundsen, A., Graff, L. S., Debernard, J. B., Gupta, A. K., He, Y., Kirkevåg, A., Schwinger, J., Tjiputra, J., Aas, K. S., Bethke, I., Fan, Y., Griesfeller, J., Grini, A., Guo, C., Ilicak, M., Karset, I. H. H., Landgren, O. A., Liakka, J., Moseid, K. O., Nummelin, A., Spensberger, C., Tang, H., Zhang, Z., Heinze, C., Iversen, T., & Schulz, M. (2019). *NCC NorESM2-MM model output prepared for CMIP6 CMIP.* Earth System Grid Federation. https://doi.org/10.22033/ESGF/CMIP6.506

Calvin, K., Bond-Lamberty, B., Clarke, L., Edmonds, J., Eom, J., Hartin, C., Kim, S., Kyle, P., Link, R., Moss, R., McJeon, H., Patel, P., Smith, S., Waldhoff, S., & Wise, M. (2017). The SSP4: A world of deepening inequality. Global Environmental Change, 42, 284–296. https://doi.org/10.1016/j.gloenvcha.2016.06.010

Collazo, S., Barrucand, M., & Rusticucci, M. (2022). Evaluation of CMIP6 models in the representation of observed extreme temperature indices trends in South America. Climatic Change, 172(1–2), 21. https://doi.org/10.1007/s10584-022-03376-1

Danabasoglu, G. (2019). NCAR CESM2 model output prepared for CMIP6 CMIP historical. China: Earth System Grid Federation. https://doi.org/10.22033/ESGF/CMIP6.7627

Dellink, R., Chateau, J., Lanzi, E., & Magné, B. (2017). Long-term economic growth projections in the Shared Socioeconomic Pathways. Global Environmental Change, 42, 200–214. https://doi.org/10.1016/j.gloenvcha.2015.06.004

Duan, Z., Liu, J., Tuo, Y., Chiogna, G., & Disse, M. (2016). Evaluation of eight high spatial resolution gridded precipitation products in Adige Basin (Italy) at multiple temporal and spatial scales. Science of The Total Environment, 573, 1536–1553. https://doi.org/10.1016/j.scitotenv.2016.08.213

Dunne, J. P., Horowitz, L. W., Adcroft, A. J., Ginoux, P., Held, I. M., John, J. G., Krasting, J. P., Malyshev, S., Naik, V., Paulot, F., Shevliakova, E., Stock, C. A., Zadeh, N., Balaji, V., Blanton, C., Dunne, K. A., Dupuis, C., Durachta, J., Dussin, R., … Zhao, M. (2020). The GFDL Earth System Model Version 4.1 (GFDL‐ESM 4.1): Overall Coupled Model Description and Simulation Characteristics. *Journal of Advances in Modeling Earth Systems*, *12*(11). https://doi.org/10.1029/2019MS002015

Fricko, O., Havlik, P., Rogelj, J., Klimont, Z., Gusti, M., Johnson, N., Kolp, P., Strubegger, M., Valin, H., Amann, M., Ermolieva, T., Forsell, N., Herrero, M., Heyes, C., Kindermann, G., Krey, V., McCollum, D. L., Obersteiner, M., Pachauri, S., … Riahi, K. (2017). The marker quantification of the Shared Socioeconomic Pathway 2: A middle-of-the-road scenario for the 21st century. Global Environmental Change, 42, 251–267. https://doi.org/10.1016/j.gloenvcha.2016.06.004

Fujimori, S., Hasegawa, T., Masui, T., Takahashi, K., Herran, D. S., Dai, H., Hijioka, Y., & Kainuma, M. (2017). SSP3: AIM implementation of Shared Socioeconomic Pathways. Global Environmental Change, 42, 268–283. https://doi.org/10.1016/j.gloenvcha.2016.06.009

Funk, C., Peterson, P., Landsfeld, M., Pedreros, D., Verdin, J., Shukla, S., Husak, G., Rowland, J., Harrison, L., Hoell, A., & Michaelsen, J. (2015). The climate hazards infrared precipitation with stations (CHIRPS): a new environmental record for monitoring extremes. Scientific Data, 2(1), 150066. https://doi.org/10.1038/sdata.2015.66

IPCC. (2022). Climate Change 2022: Impacts, Adaptation and Vulnerability. Retrieved from https://report.ipcc.ch/ar6/wg2/IPCC_AR6_WGII_FullReport.pdf

Jiang, L., & O’Neill, B. C. (2017). Global urbanization projections for the Shared Socioeconomic Pathways. Global Environmental Change, 42, 193–199. https://doi.org/10.1016/j.gloenvcha.2015.03.008

Jungclaus, J., Bittner, M., Wieners, K.-H., Wachsmann, F., Schupfner, M., Legutke, S., Giorgetta, M., Reick, C., Gayler, V., Haak, H., de Vrese, P., Raddatz, T., Esch, M., Mauritsen, T., von Storch, J.-S., Behrens, J., Brovkin, V., Claussen, M., Crueger, T., Fast, I., Fiedler, S., Hagemann, S., Hohenegger, C., Jahns, T., Kloster, S., Kinne, S., Lasslop, G., Kornblueh, L., Marotzke, J., Matei, D., Meraner, K., Mikolajewicz, U., Modali, K., Müller, W., Nabel, J., Notz, D., Peters-von Gehlen, K., Pincus, R., Pohlmann, H., Pongratz, J., Rast, S., Schmidt, H., Schnur, R., Schulzweida, U., Six, K., Stevens, B., Voigt, A., & Roeckner, E. (2019). *MPI-M MPI-ESM1-2-HR model output prepared for CMIP6 CMIP historical.* Earth System Grid Federation. https://doi.org/10.22033/ESGF/CMIP6.6594

Katsanos, D., Retalis, A., & Michaelides, S. (2016). Validation of a high-resolution precipitation database (CHIRPS) over Cyprus for a 30-year period. Atmospheric Research, 169, 459–464. https://doi.org/10.1016/j.atmosres.2015.05.015

Kc, S., & Lutz, W. (2017). The human core of the shared socioeconomic pathways: Population scenarios by age, sex and level of education for all countries to 2100. Global Environmental Change, 42, 181–192. https://doi.org/10.1016/j.gloenvcha.2014.06.004

Krasting, J. P., John, J. G., Blanton, C., McHugh, C., Nikonov, S., Radhakrishnan, A., Rand, K., Zadeh, N. T., Balaji, V., Durachta, J., Dupuis, C., Menzel, R., Robinson, T., Underwood, S., Vahlenkamp, H., Dunne, K. A., Gauthier, P. P. G., Ginoux, P., Griffies, S. M., Hallberg, R., Harrison, M., Hurlin, W., Malyshev, S., Naik, V., Paulot, F., Paynter, D. J., Ploshay, J., Reichl, B. G., Schwarzkopf, D. M., Seman, C. J., Silvers, L., Wyman, B., Zeng, Y., Adcroft, A., Dunne, J. P., Dussin, R., Guo, H., He, J., Held, I. M., Horowitz, L. W., Lin, P., Milly, P. C. D., Shevliakova, E., Stock, C., Winton, M., Wittenberg, A. T., Xie, Y., & Zhao, M. (2018). *NOAA-GFDL GFDL-ESM4 model output prepared for CMIP6 CMIP.* Earth System Grid Federation. https://doi.org/10.22033/ESGF/CMIP6.1407

Kriegler, E., Bauer, N., Popp, A., Humpenöder, F., Leimbach, M., Strefler, J., Baumstark, L., Bodirsky, B. L., Hilaire, J., Klein, D., Mouratiadou, I., Weindl, I., Bertram, C., Dietrich, J.-P., Luderer, G., Pehl, M., Pietzcker, R., Piontek, F., Lotze-Campen, H., … Edenhofer, O. (2017). Fossil-fueled development (SSP5): An energy and resource intensive scenario for the 21st century. Global Environmental Change, 42, 297–315. https://doi.org/10.1016/j.gloenvcha.2016.05.015

Lee, W.-L., & Liang, H.-C. (2022). CMIP6.CMIP.AS-RCEC.TaiESM1.historical. https://doi.org/10.22033/ESGF/CMIP6.9755

Lovato, T., Peano, D., Butenschön, M. (2021). *CMCC CMCC-ESM2 model output prepared for CMIP6 ScenarioMIP.* Earth System Grid Federation. https://doi.org/10.22033/ESGF/CMIP6.13168

Martens, B., Miralles, D. G., Lievens, H., van der Schalie, R., de Jeu, R. A. M., Fernández-Prieto, D., Beck, H. E., Dorigo, W. A., & Verhoest, N. E. C. (2017). GLEAM v3: satellite-based land evaporation and root-zone soil moisture. Geoscientific Model Development, 10(5), 1903–1925. https://doi.org/10.5194/gmd-10-1903-2017

Masui, T., Matsumoto, K., Hijioka, Y., Kinoshita, T., Nozawa, T., Ishiwatari, S., Kato, E., Shukla, P. R., Yamagata, Y., & Kainuma, M. (2011). An emission pathway for stabilization at 6 Wm−2 radiative forcing. Climatic Change, 109(1–2), 59–76. https://doi.org/10.1007/s10584-011-0150-5

McNally, A., Arsenault, K., Kumar, S., Shukla, S., Peterson, P., Wang, S., Funk, C., Peters-Lidard, C. D., & Verdin, J. P. (2017). A land data assimilation system for sub-Saharan Africa food and water security applications. Scientific Data, 4(1), 170012. https://doi.org/10.1038/sdata.2017.12

Miralles, D. G., Holmes, T. R. H., de Jeu, R. A. M., Gash, J. H., Meesters, A. G. C. A., & Dolman, A. J. (2011). Global land-surface evaporation estimated from satellite-based observations. Hydrology and Earth System Sciences, 15(2), 453–469. https://doi.org/10.5194/hess-15-453-2011

Moss, R. H., Edmonds, J. A., Hibbard, K. A., Manning, M. R., Rose, S. K., van Vuuren, D. P., Carter, T. R., Emori, S., Kainuma, M., Kram, T., Meehl, G. A., Mitchell, J. F. B., Nakicenovic, N., Riahi, K., Smith, S. J., Stouffer, R. J., Thomson, A. M., Weyant, J. P., & Wilbanks, T. J. (2010). The next generation of scenarios for climate change research and assessment. Nature, 463(7282), 747–756. https://doi.org/10.1038/nature08823

Mu, Q., Zhao, M., & Running, S. W. (2011)a. MODIS Global Terrestrial Evapotranspiration (ET) Product (MOD16A2). NASA MODIS Adaptive Processing System, Goddard Space Flight Center. https://modis.gsfc.nasa.gov

Mu, Q., Zhao, M., & Running, S. W. (2011)b. Improvements to a MODIS global terrestrial evapotranspiration algorithm. Remote Sensing of Environment, 115(8), 1781–1800. https://doi.org/10.1016/j.rse.2011.02.019

O’Neill, B. C., Carter, T. R., Ebi, K., Harrison, P. A., Kemp-Benedict, E., Kok, K., Kriegler, E., Preston, B. L., Riahi, K., Sillmann, J., van Ruijven, B. J., van Vuuren, D., Carlisle, D., Conde, C., Fuglestvedt, J., Green, C., Hasegawa, T., Leininger, J., Monteith, S., & Pichs-Madruga, R. (2020). Achievements and needs for the climate change scenario framework. Nature Climate Change, 10(12), 1074–1084. https://doi.org/10.1038/s41558-020-00952-0

O’Neill, B. C., Kriegler, E., Ebi, K. L., Kemp-Benedict, E., Riahi, K., Rothman, D. S., van Ruijven, B. J., van Vuuren, D. P., Birkmann, J., Kok, K., Levy, M., & Solecki, W. (2017). The roads ahead: Narratives for shared socioeconomic pathways describing world futures in the 21st century. Global Environmental Change, 42, 169–180. https://doi.org/10.1016/j.gloenvcha.2015.01.004

O’Neill, B. C., Tebaldi, C., van Vuuren, D. P., Eyring, V., Friedlingstein, P., Hurtt, G., Knutti, R., Kriegler, E., Lamarque, J.-F., Lowe, J., Meehl, G. A., Moss, R., Riahi, K., & Sanderson, B. M. (2016). The Scenario Model Intercomparison Project (ScenarioMIP) for CMIP6. Geoscientific Model Development, 9(9), 3461–3482. https://doi.org/10.5194/gmd-9-3461-2016

Perdigón‐Morales, J., Romero‐Centeno, R., Pérez, P. O., & Barrett, B. S. (2018). The midsummer drought in Mexico: perspectives on duration and intensity from the CHIRPS precipitation database. International Journal of Climatology, 38(5), 2174–2186. https://doi.org/10.1002/joc.5322

Riahi, K., Rao, S., Krey, V., Cho, C., Chirkov, V., Fischer, G., Kindermann, G., Nakicenovic, N., & Rafaj, P. (2011). RCP 8.5—A scenario of comparatively high greenhouse gas emissions. Climatic Change, 109(1–2), 33–57. https://doi.org/10.1007/s10584-011-0149-y

Riahi, K., van Vuuren, D. P., Kriegler, E., Edmonds, J., O’Neill, B. C., Fujimori, S., Bauer, N., Calvin, K., Dellink, R., Fricko, O., Lutz, W., Popp, A., Cuaresma, J. C., KC, S., Leimbach, M., Jiang, L., Kram, T., Rao, S., Emmerling, J., … Tavoni, M. (2017). The Shared Socioeconomic Pathways and their energy, land use, and greenhouse gas emissions implications: An overview. Global Environmental Change, 42, 153–168. https://doi.org/10.1016/j.gloenvcha.2016.05.009

Rodell, M., Houser, P. R., Jambor, U., Gottschalck, J., Mitchell, K., Meng, C.-J., Arsenault, K., Cosgrove, B., Radakovich, J., Bosilovich, M., Entin, J. K., Walker, J. P., Lohmann, D., & Toll, D. (2004). The Global Land Data Assimilation System. Bulletin of the American Meteorological Society, 85(3), 381–394. https://doi.org/10.1175/BAMS-85-3-381

Song, Z., Qiao, F., Bao, Y., Shu, Q., Song, Y., & Yang, X. (2019). *FIO-QLNM FIO-ESM2.0 model output prepared for CMIP6 ScenarioMIP ssp245*. Earth System Grid Federation. https://doi.org/10.22033/ESGF/CMIP6.9209

Thomson, A. M., Calvin, K. v., Smith, S. J., Kyle, G. P., Volke, A., Patel, P., Delgado-Arias, S., Bond-Lamberty, B., Wise, M. A., Clarke, L. E., & Edmonds, J. A. (2011). RCP4.5: a pathway for stabilization of radiative forcing by 2100. Climatic Change, 109(1–2), 77–94. https://doi.org/10.1007/s10584-011-0151-4

van Vuuren, D. P., den Elzen, M. G. J., Lucas, P. L., Eickhout, B., Strengers, B. J., van Ruijven, B., Wonink, S., & van Houdt, R. (2007). Stabilizing greenhouse gas concentrations at low levels: an assessment of reduction strategies and costs. Climatic Change, 81(2), 119–159. https://doi.org/10.1007/s10584-006-9172-9

van Vuuren, D. P., Edmonds, J., Kainuma, M., Riahi, K., Thomson, A., Hibbard, K., Hurtt, G. C., Kram, T., Krey, V., Lamarque, J.-F., Masui, T., Meinshausen, M., Nakicenovic, N., Smith, S. J., & Rose, S. K. (2011). The representative concentration pathways: an overview. Climatic Change, 109(1–2), 5–31. https://doi.org/10.1007/s10584-011-0148-z

van Vuuren, D. P., Stehfest, E., Gernaat, D. E. H. J., Doelman, J. C., van den Berg, M., Harmsen, M., de Boer, H. S., Bouwman, L. F., Daioglou, V., Edelenbosch, O. Y., Girod, B., Kram, T., Lassaletta, L., Lucas, P. L., van Meijl, H., Müller, C., van Ruijven, B. J., van der Sluis, S., & Tabeau, A. (2017). Energy, land-use and greenhouse gas emissions trajectories under a green growth paradigm. Global Environmental Change, 42, 237–250. https://doi.org/10.1016/j.gloenvcha.2016.05.00

Wieners, K.-H., Giorgetta, M., Jungclaus, J., Reick, C., Esch, M., Bittner, M., et al. (2022). CMIP6.CMIP.MPI-M.MPI-ESM1-2-LR.historical. https://doi.org/10.22033/ESGF/CMIP6.6595

Wyser, K., van Noije, T., Yang, S., von Hardenberg, J., O’Donnell, D., & Döscher, R. (2020). On the increased climate sensitivity in the EC-Earth model from CMIP5 to CMIP6. Geoscientific Model Development, 13(8), 3465–3474. https://doi.org/10.5194/gmd-13-3465-2020

Xin, X., Zhang, J., Zhang, F., Wu, T., Shi, X., Li, J., Chu, M., Liu, Q., Yan, J., Ma, Q., & Wei, M. (2018). *BCC BCC-CSM2MR model output prepared for CMIP6 CMIP*. Earth System Grid Federation. https://doi.org/10.22033/ESGF/CMIP6.1725

Yukimoto, S., Koshiro, T., Kawai, H., Oshima, N., Yoshida, K., Urakawa, S., Tsujino, H., Deushi, M., Tanaka, T., Hosaka, M., Yoshimura, H., Shindo, E., Mizuta, R., Ishii, M., Obata, A., & Adachi, Y. (2019). *MRI MRI-ESM2.0 model output prepared for CMIP6 CMIP*. Earth System Grid Federation. https://doi.org/10.22033/ESGF/CMIP6.621
